# Stimulus specific cortical activity associated with ignoring distraction during working memory encoding and maintenance

**DOI:** 10.1038/s41598-023-34967-2

**Published:** 2023-06-02

**Authors:** Charlotte Ashton, Andre D. Gouws, Marcus Glennon, Abhishek Das, Yit-Keat Chen, Charlotte Chrisp, Ismail Felek, Theodore P. Zanto, Fiona McNab

**Affiliations:** 1 Department of Psychology, University of York, York, YO10 5DD UK; 2 York Neuroimaging Centre, University of York, York, YO10 5NY UK; 3 Department of Neurology, University of California San Francisco, San Francisco, 94158 USA

**Keywords:** Neuroscience, Psychology

## Abstract

Distraction disrupts Working Memory (WM) performance, but how the brain filters distraction is not known. One possibility is that neural activity associated with distractions is suppressed relative to a baseline/passive task (biased competition). Alternatively, distraction may be denied access to WM, with no suppression. Furthermore, behavioural work indicates separate mechanisms for ignoring distractions which occur (1) while we put information into WM (Encoding Distraction, ED) and (2) while we maintain already encoded information during the WM delay period (Delay Distraction, DD). Here we used fMRI in humans to measure category-sensitive cortical activity and probe the extent to which ED/DD mechanisms involve enhancement/suppression during a WM task. We observed significant enhancement of task-relevant activity, relative to a passive view task, which did not differ according to whether or when distractors appeared. For both ED and DD we found no evidence of suppression, but instead a robust increase in stimulus specific activity in response to additional stimuli presented during the passive view task, which was not seen for the WM task, when those additional stimuli were to be ignored. The results indicate that ED/DD resistance does not necessarily involve suppression of distractor-related activity. Rather, a rise in distractor-associated activity is prevented when distractors are presented, supporting models of input gating, and providing a potential mechanism by which input-gating might be achieved.

## Introduction

Our ability to effectively exclude distraction and selectively attend to relevant stimuli has been identified as a basis for Working Memory Capacity (WMC) limitations in younger adults, older adults and in patients^1–4^. The mechanisms for distractor resistance are not yet well understood^5^. On the one hand, there is research that describes a process of top-down inhibition, whereby the sensory processing of distractors is reduced, or suppressed and a process of enhancement, whereby sensory processing of relevant information is increased^6,7^, see also^5,8^. According to this biased-competition view, activation for task relevant/irrelevant information may be selectively up or down weighted, so that irrelevant information is less likely to meet a threshold for entry into Working Memory (WM).

On the other hand, other work describes a process of “input gating”, by which control processes block task-irrelevant information from being encoded into WM, and allow access only to task-relevant information^9,10^. Rather than suppressing activation associated with distractors, according to this view the gate allowing access to WM can be switched to open or closed, and when closed sensory input is prohibited from modifying the contents of WM (see also^5^). Furthermore, it is not yet known how such gating may be achieved. Increases in category-sensitive activity when scenes/faces are displayed are robust^11–13^. One possibility is that gating may selectively attenuate this increase for stimuli that should be ignored. Alternatively, gating from WM may be implemented at a later stage.

Here we used fMRI to investigate whether the modulation of category-sensitive activity, within the context of a WM task, supports a biased competition account of distractor resistance, or an input gating account. Using fMRI there have been reports of “enhancement” of activity in stimulus-specific visual processing regions associated with stimuli that should be remembered, and “suppression” of activity associated with stimuli that should be ignored, relative to a stimulus-matched passively viewed baseline^14–17^. This data supports the biased competition account of distractor resistance. Furthermore, it has been shown that a reduction in this suppression (but not enhancement) may contribute to impaired WM in healthy ageing^18–20^ as well as the over-processing of distractors during high WM load conditions^17^. The specific effects of aging on suppression indicates enhancement and suppression operate as distinct mechanisms and further support the biased competition account of distractor resistance.

However, it is not clear whether features of the tasks used in those studies may have accounted for the findings of suppression. For example, some of those studies involved sequential presentation of task-relevant and irrelevant stimuli, in a randomized order, with task relevance only becoming apparent when each stimulus is displayed^14,18,21^. Others used sequential presentation of superimposed face and scene stimuli, which requires the participant to switch between “seeing” a face or a scene. Another approach has been to ask participants to initially remember all the stimuli, and then subsequently forget a subset^22^. Here we revisit those findings of distractor suppression and address whether suppression can also be seen with simultaneous presentation of spatially-distinct relevant and irrelevant stimuli—compared to a stimulus matched passively viewed condition. Alternatively, if activation of task-irrelevant stimuli is equivalent to activation of those stimuli under passive view task conditions, this would be more closely aligned to an input-gating view of distractor-resistance.

Separate behavioral work has identified two potential bases for our limited WMC: a person’s ability to ignore irrelevant stimuli that appear with the stimuli that should be remembered (during WM encoding; “Encoding Distraction”, ED) and their ability to ignore irrelevant stimuli presented during the WM delay period, when relevant stimuli are held in mind but no longer physically present (“Delay Distraction”, DD)^23^. Research indicates that our ability to ignore ED and DD uniquely predict WMC^23^ and are differentially affected by ageing^24^, suggesting separate mechanisms. We assessed both ED and DD conditions, and addressed whether activity in stimulus-specific visual processing regions is suppressed when those stimuli are to be ignored during WM encoding (ED) or ignored during WM maintenance (DD).

Our approach was therefore to directly assess the magnitude of enhancement (attend > passive) and suppression (ignore < passive) of activity in stimulus-specific visual processing regions while distractors are presented either during encoding (ED), during the delay (DD), or not at all (No Distraction (ND) control). We were particularly interested in whether both enhancement and suppression would be observed (as predicted by the biased competition account) or whether we would observe enhancement but not suppression (as predicted by an input-gating account).

## Methods

The methods were approved by York Neuroimaging Centre Research Ethics Committee at the University of York, UK. All methods were performed in accordance with the relevant guidelines and regulations. The participants provided written informed consent to take part in the study.

### Participants

Thirty-seven healthy participants (right handed) participated in the study. Datasets were excluded when accuracy was equal to or below 50% for any block (7 participants). After collecting data from 21 participants, we added practice trials prior to scanning, and feedback on accuracy after each block, after which no further participants met the exclusion criteria. Data from three further participants were omitted due to a technical error displaying the stimuli or excessive movement. fMRI data from 27 participants (14 females, ages 18–29) were analysed.

Due to concerns that participants might close their eyes during the WM delay in order to avoid seeing the Delay Distraction (DD), we also conducted a separate experiment, using the same paradigm, but with different participants, in which we examined the number of eye-closures in the different conditions. 24 participants took part in that experiment (18 females, mean age = 19.9 years, standard deviation = 2.2 years).

### Tasks and stimuli

**Figure 1:**
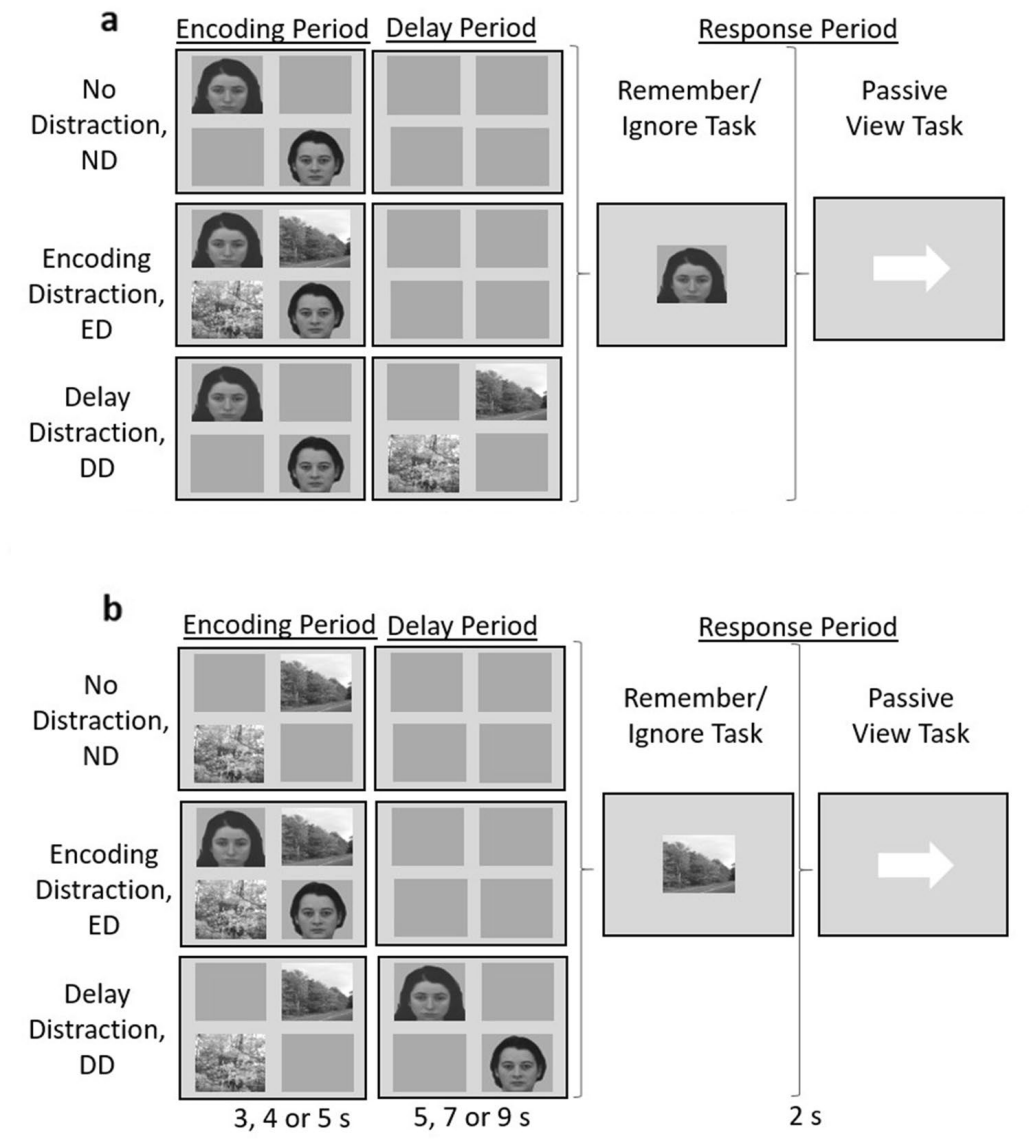
The paradigm. The three distraction conditions (No Distraction, Encoding Distraction and Delay Distraction) for the working memory task, where faces (**a**) or scenes (**b**) were to be remembered and scenes (**a**) or faces (**b**) were to be ignored, and the stimulus-matched passive view task. Each combination of distraction condition and task was presented in a separate block (note that the ED passive view combination is shown in both a and b, but as the stimuli and task were identical, only one block was given, so there were 11 blocks in total).

We used face and scene stimuli to examine cortical activity associated with remembering task-relevant stimuli (targets) and ignoring task-irrelevant stimuli (distractors) during a WM task with No overt Distraction (ND), Encoding Distraction (ED), or with Delay Distraction (DD) (Fig. 1). In the ND condition only target stimuli were displayed. In the ED condition both targets and distractors were displayed during the WM encoding period, and no stimuli were shown during the WM delay period. In the DD condition, target stimuli were shown during the encoding period (i.e., shown first) and distractors were shown during the WM delay period (i.e., shown second). We compared each of these conditions to a separate passive view task, which involved identical presentation of stimuli, but for this task none of the stimuli were to be remembered (Fig. 1). Therefore there was a passive view task that was matched to the ED condition, where both faces and scenes were shown simultaneously, a passive view task that was matched to the DD condition in which faces were shown first, followed by scenes, and another passive view task that was also matched to the DD condition in which scenes were shown first, followed by faces. There were also two passive view conditions matched to each of the ND conditions (i.e., with only faces shown or with only scenes shown).

Greyscale images of natural scenes and faces with neutral expressions were used (these images had also been used by Gazzaley et al.^14^ and informed consent was obtained for the publication of identifying images in an online open-access publication). For each of the 11 blocks, the same set of face and scene images (subtending a visual angle of 6.92° horizontally and 6.46° vertically) were used, with no repetitions within a block. For each block there were two targets, each presented in a pseudo-randomly selected quadrant of the screen. When disractors were shown they were in the remaining quadrants. All other quadrants were grey (Fig. 1). For the remember/ignore task, a single, centrally presented image from the target stimulus category was shown during the response period, prompting the participant to indicate, via a button press, whether it matched one of the targets from the encoding period, which it did for half of the trials. For the passive view task, participants were asked to indicate whether an arrow pointed to the left (which it did for half the trials) or to the right. For each block there were 24 of the trials described, and an additional six trials in which a white fixation cross was presented instead of a delay period, to help de-correlate the regressors associated with the delay period from regressors associated with other parts of the trial.

A functional localizer was also conducted to identify stimulus-specific (face/scene) visual processing regions. For the functional localiser scan, centrally presented single images (112 faces and 112 scenes) were presented sequentially for 1.4 s each. Face and scene blocks (each with 16 images) were presented in pairs, with 22.4 s in between each pair. Participants performed a 1-back task, pressing a button if they identified a match (which there was for 28 images).

### Procedure

Each participant completed two scanning sessions on separate days. Each condition was presented in a separate scanning run. The first session included the ND blocks for each task and stimulus type (four blocks, with the order counterbalanced) as well as the functional localiser scan. The second session included the remaining seven blocks, with order counterbalanced. Prior to entering the scan room, participants completed different visuo-spatial WM tasks which are not reported here.

### MRI acquisition

Imaging data were collected using a 3.0 Tesla MRI scanner (Siemens) with a 20-channel phased-array head coil and a bottom-up interleaved Echo Planar Imaging (EPI) sequence. For the experimental scan: TR = 2.1 s, TE = 30 ms, flip angle = 80°, FOV = 192 × 192 × 108 mm, matrix size = 64 × 64, number of volumes = 213, voxel size = 3 × 3 mm, slice thickness = 3 mm with no inter-slice gap and 36 slices were acquired. For the functional localiser scan: TR = 2 s, TE = 30 ms, flip angle = 80°, FOV = 192 × 192 × 108 mm, matrix size = 64 × 64, number of volumes = 227, voxel size = 3 × 3 mm, slice thickness = 3 mm with no inter-slice gap and 36 slices were acquired. A T1-weighted structural image was obtained for each participant (TR = 2.3 s, TE = 2.26 ms, flip angle = 8°, FOV = 256 × 256, voxel size = 1 × 1, slice thickness = 1 mm, number of slices = 176) and a T1-weighted FLAIR image (TR = 3 s, TE = 8.6 ms, flip angle = 150°, FOV = 192 × 192 mm, matrix size = 256 × 256, voxel size = 0.75 × 0.75 mm, slice thickness = 3.0 mm, number of slices = 36) was taken in the same plane as the EPI data, for co-registration of the functional data to the structural image.

### Data pre-processing

For data processing, FEAT (FMRI Expert Analysis Tool) version 6.0, part of FSL FMRIB’s Software Library, (www.fmrib.ox.ac.uk/fsl) was used. The data were skull stripped using BET (Brain Extraction Tool^25^), motion corrected using MCFLIRT^26^, slice time corrected using fourier-space–time-series phase-shifting and non-brain removal using BET^25^ and spatially smoothed using a Gaussian Kernel of FWHM (full width at half maximum) 8 mm and high pass temporal filtering (Gaussian-weighted least-squares straight line fitting, with sigma = 100 s). Registration of the fMRI data to the structural data and standard brain (MNI152 2 mm) was carried out using FLIRT^26,27^. Registration from structural data to the standard brain was further refined using FNIRT^28,29^.

A time-series statistical analysis was carried out using FILM with local autocorrelation correction^30^. The general linear model included regressors for the encoding period (3, 4 or 5 s), delay period (5, 7 or 9 s), and the response period (2 s). Only correct trials were analysed and there was an additional regressor for incorrect trials. Regressors were convolved with a single gamma hemodynamic response function.

Scene-sensitive PPA ROIs were defined for each hemisphere using data from the functional localiser scan, and the contrast: scenes > faces. Face-sensitive FFA ROIs were also defined using the contrast: faces > scenes, and data from those ROIs are reported in the Supplementary Materials. For each contrast the maximally active voxel within the respective anatomical region, in each participant’s native brain space, was selected. Two voxels were added to the peak voxel in the x, y and z directions, making a ROI of 7 contiguous voxels. For each time period, and each block we extracted the parameter estimates using featquery (FSL 5.0.1).

### Statistical analysis

In line with the previous studies which found Left PPA (LPPA) to give a “robust marker of modulation”^18^ (also see the Supplementary Material for details of comparisons between remembering versus ignoring the face/scene stimuli), and therefore focused on this region. We report data from LPPA here, and report data from right PPA, left FFA and right FFA in the Supplementary Material. Our approach was to use activity associated with scene stimuli as a tool to investigate the modulation of cortical activity based on the allocation of attention. As such, our focus was on the effects of task (remember, ignore or passive view) and interactions between task and distraction. All of our analyses involved repeated measures ANOVAs and follow-up paired t-tests. Where Mauchley’s test of sphericity indicated that the assumption of sphericity had been violated, the Greenhouse Geisser statistic is reported. The data were analysed using IBM SPSS Statistics version 25. The accompanying P values were determined by two tailed analysis and considered significant if p < 0.05.

### In-scanner performance

Percentage accuracy was calculated for each condition (ND, ED, DD) of each task (passive view, WM). Behavioral responses during scanning were also used to establish how much information was held in WM (WMC) for each task and distraction condition, using the standard formula^1,31^ *K* = *S (H – F)*, where *K* is working memory capacity, *S* is the array size (in this case 2), *H* is the observed hit rate and *F* is the false alarm rate. For both face and scene stimuli, repeated measures ANOVAs, with follow-up paired t-tests, were used to examine the effect of task and condition on performance.

### Eye-closure experiment

Despite impaired performance associated with the DD condition, and the varying duration of the delay period, we were concerned that participants may avoid seeing the DD by closing their eyes during the delay period. Therefore, using different participants we examined eye closures during the delay period of the task. We compared the number of eye-closures and the duration of eye-closures between ND and DD conditions using a repeated measures ANOVA with factors condition (ND or DD) and stimulus type (faces or scenes).

## Results

### In-scanner performance

Accuracy in the scanner was high (Table 1). As anticipated, for the passive view task there was no difference in accuracy between the different conditions or between the different stimulus types (main effect of condition: F(1.398,36.359) = 0.10, p = 0.909 (Greenhouse–Geisser was used as W(2) = 0.57, p < 0.001), main effect of stimulus type: F(1, 26) = 0.04, p = 0.852, condition*stimulus type interaction: F(1.608,41.815) = 0.02, p = 0.969 (Greenhouse–Geisser was used as W(2) = 0.76, p = 0.031).

**Table 1:** Task performance.

The stimuli to remember in the WM task	WM task						Passive view task					
	Faces			Scenes			Faces			Scenes		
Condition	ND	ED	DD	ND	ED	DD	ND	ED	DD	ND	ED	DD
Accuracy	93.70 (5.94)	92.90 (7.57)	91.33 (6.19)	84.14 (7.76)	88.49 (8.37)	79.97 (10.31)	98.12 (4.27)	97.72 (5.20)	98.21 (3.09)	97.99 (3.90)	97.72 (5.20)	98.09 (3.29)
WMC estimate	1.82 (0.19)	1.79 (0.24)	1.78 (0.21)	1.46 (0.37)	1.62 (0.35)	1.33 (0.36)						

Mean accuracy (% correct) and WMC estimate (K value) for each task, stimulus type and condition. Standard deviation is shown in brackets.

For the WM task, estimates of WMC (K values) significantly differed between conditions and types of stimuli (condition*stimulus type interaction: F(2,52) = 4.30, p = 0.019). For faces there was no significant difference between conditions (F(2, 52) = 0.96, p = 0.389). For scenes there was (F(2,52) = 9.76, p < 0.001), with significantly higher WMC for ED compared to ND (t(26) = − 3.20, p = 0.004) and significantly lower WMC for DD compared to ED (t(26) = 4.10, p < 0.001), but no significant difference between ND and DD conditions (t(26) = 1.86, p = 0.074).

### Assessment of enhancement and suppression: ED

**Figure 2:**
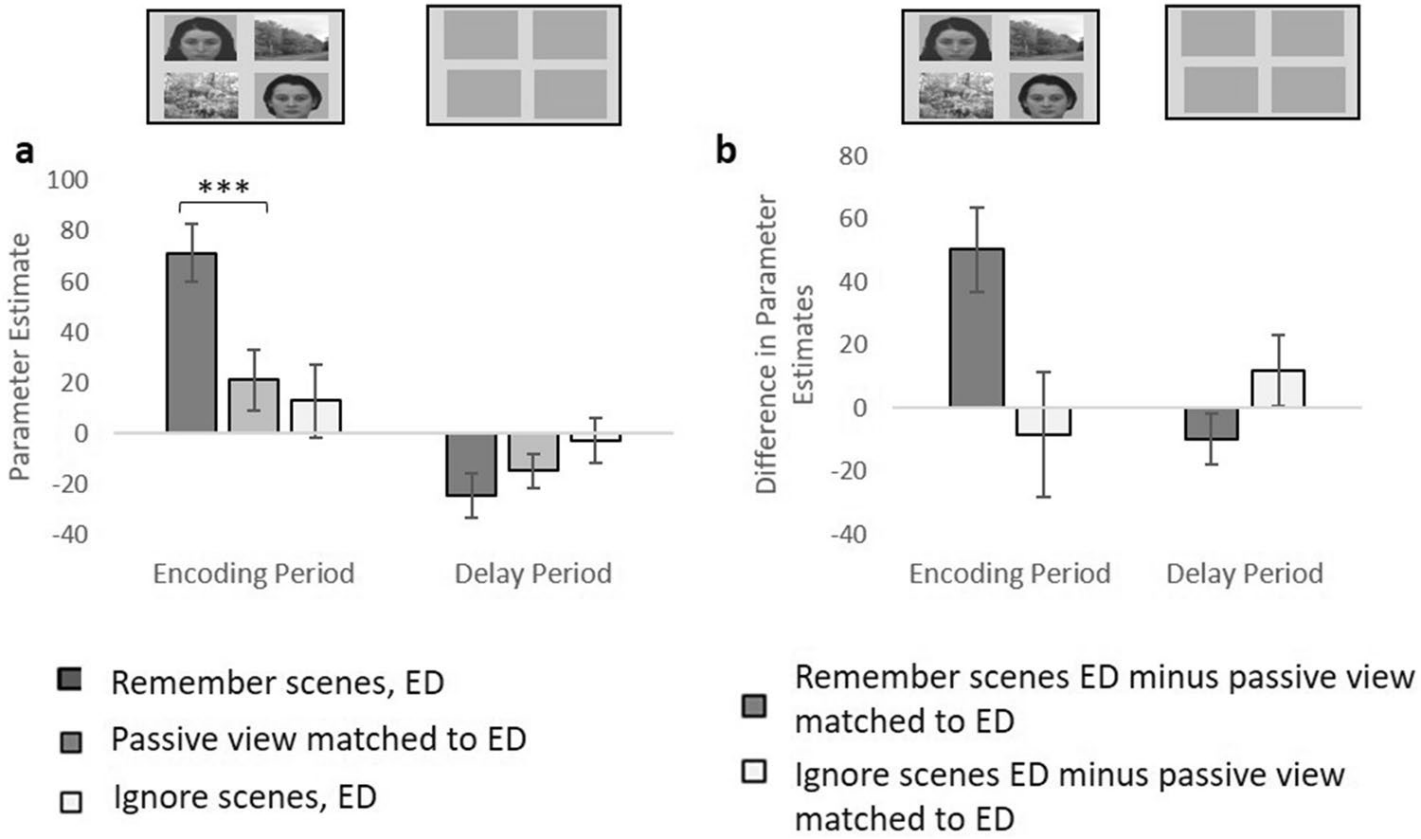
LPPA activity associated with remembering and ignoring scenes during the ED condition, compared to the stimulus-matched passive view condition. (**a**) LPPA activity for the ED condition when the task was to remember scenes/ignore faces, passively view scenes and faces, and remember faces/ignore scenes, for the encoding and delay period. The stimuli displayed for each time period are shown at the top of the figure. ***p < 0.001. For illustration, we subtracted passive view activity from each of the other two task conditions, to show the level of enhancement and suppression (**b**). The error bars indicate s.e.m.

First, for the ED condition, we assessed whether LPPA activity was enhanced when scenes should be remembered, and/or suppressed when scenes should be ignored, relative the stimulus-matched passive view baseline conditions. With repeated measures ANOVAs and paired t-tests we first examined enhancement, considering the effects of time period (encoding, delay) and task (remember, passive view). There was a significant interaction between time period and task (F(1,26) = 13.88, p < 0.001). Whereas there was significant enhancement during the encoding period (t(26) = 3.71, p < 0.001), for the delay period there was no significant difference between tasks (t(26) = − 1.25, p = 0.221) (Fig. 2). Of note, RPPA also exhibited enhancement (see Supplementary Material).

We then considered suppression during the ED condition, looking at the effects of time period and task (ignore, passive view). There was no significant task difference (F(1,26) = 0.02, p = 0.899; Fig. 2) and no significant task*time period interaction (F(1,26) = 1.63, p = 0.213). There was only a significant main effect of time period (F(1,26) = 9.32, p = 0.005). Similar results were observed for RPPA (see Supplementary Material). Therefore, there was no evidence for suppression of PPA activity when ignoring scenes during the ED condition compared to the stimulus-matched passive view condition.

### Assessment of enhancement and suppression: DD

**Figure 3:**
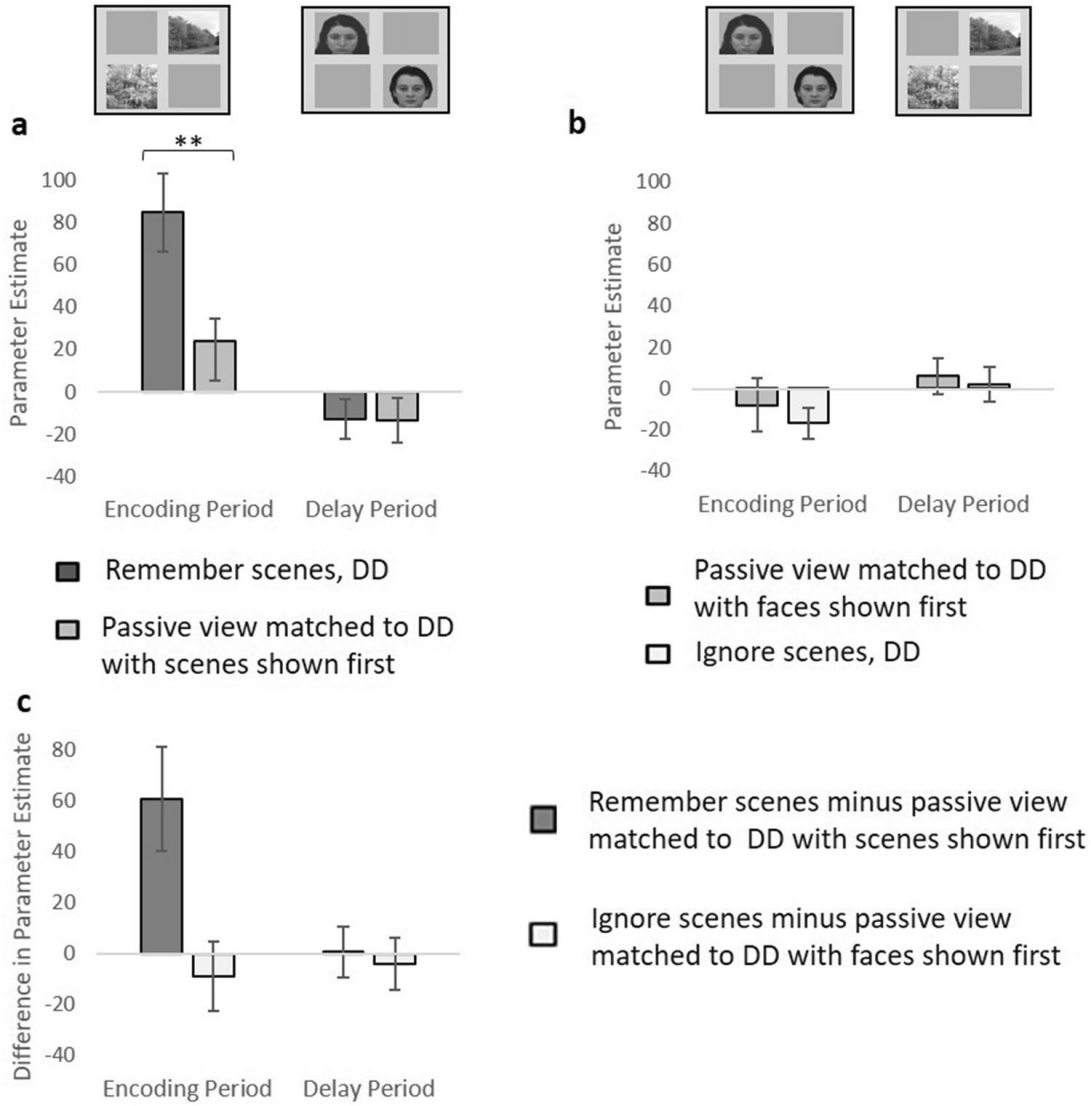
LPPA activity associated with remembering and ignoring scenes during the DD condition, compared to the stimulus-matched passive view conditions. LPPA activity for the DD condition when the task was to (**a**) remember scenes presented during the encoding period and ignore faces presented during the delay period or passively view scenes during the “encoding period” and then passively view faces during the “delay period”, (**b**) passively view faces during the “encoding period” and then passively view scenes during the “delay period”, and remember faces presented during the encoding period and ignore scenes presented during the delay period. The stimuli displayed for each time period are shown at the top of the figures. **p < 0.01. For illustration, we subtracted passive view activity from its stimulus-matched remember/ignore task condition, to show the level of enhancement and suppression (**c**). The error bars indicate s.e.m.

To investigate enhancement for DD, we compared the remember scenes condition with its stimulus-matched passive view condition. There was a significant interaction between time period and task (F(1,26) = 9.85, p = 0.004, Fig. 3a and c). Paired t-tests identified significant enhancement during the encoding period (t(26) = 2.97, p = 0.006) but no significant difference between tasks during the delay period (t(26) = 0.08, p = 0.940). Similar results were observed for RPPA (see Supplementary Material). Therefore, as with ED, the DD condition exhibited significant enhancement only during the encoding period.

To investigate suppression due to DD, we compared ignore scenes (DD) versus the stimulus-matched passive view task, and the results are shown in Fig. 3a and c. There was no significant main effect of task (F(1,26) = 0.336, p = 0.567) and no significant interaction between time period and task (F(1,26) = 0.27, p = 0.611). There was only a significant main effect of time period (F(1,26) = 8.82, p = 0.006). Therefore, as with ED, for DD there was no evidence of significantly lower activity when ignoring DD compared to passively viewing the same stimuli.

### Effects of distraction on measures of enhancement

**Figure 4:**
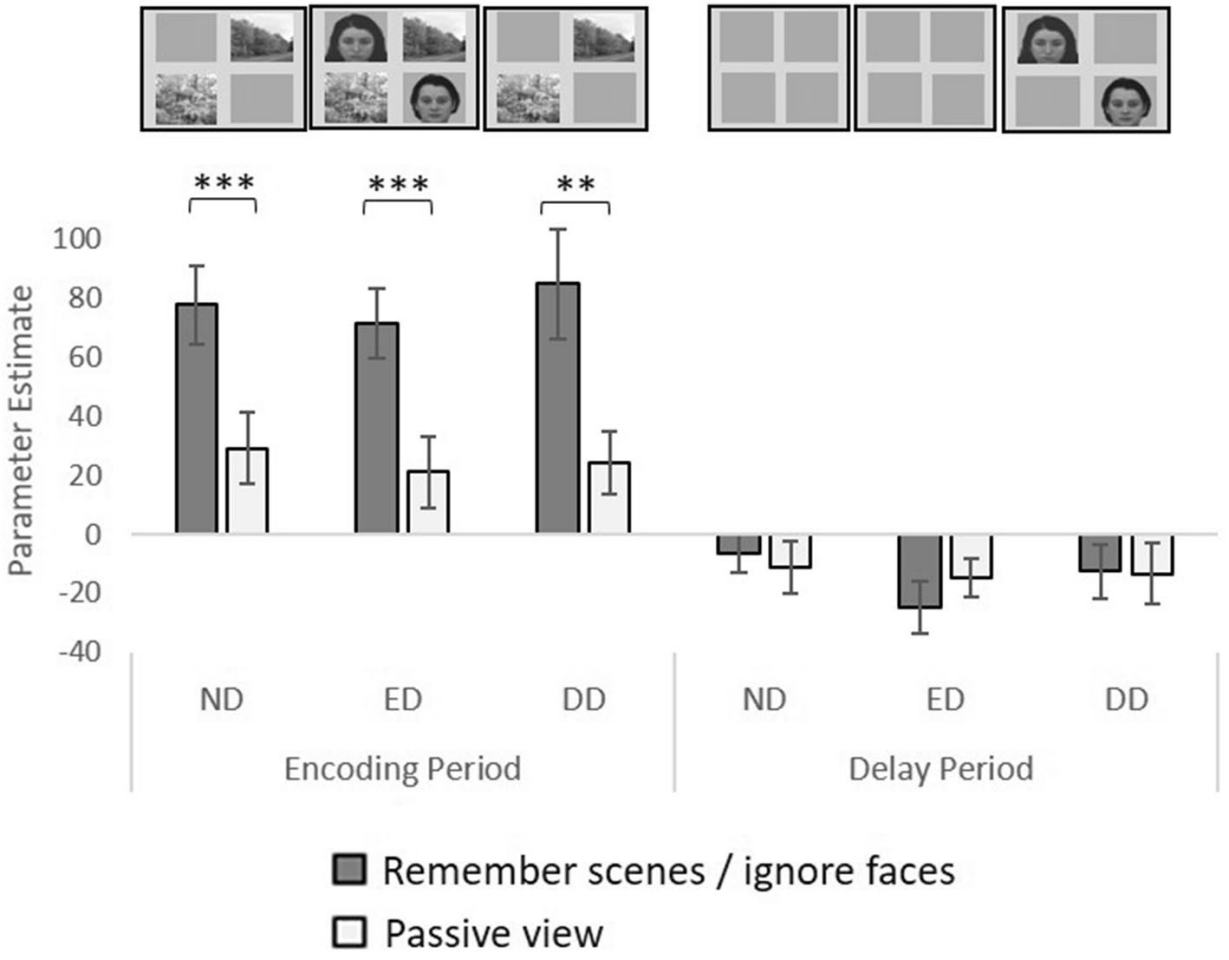
LPPA activity associated with remembering scenes during the ND, ED and DD conditions, compared to the stimulus-matched passive view conditions. (**a**) LPPA activity when scenes should be remembered and faces (when displayed) should be ignored for the ND, ED and DD conditions, and also for the stimulus-matched passive view task, for both time periods. The error bars indicate s.e.m. **p < 0.01 and ***p < 0.001.

The analyses above demonstrated enhancement, but not suppression, of activity within stimulus-specific regions during the WM tasks. This appears to support the input-gating account of distraction. However, another possibility is that there is greater target-enhancement when distractors are present, compared to when distractors are absent, which would be more in line with a biased-competition account of distractor resistance. To interrogate this possibility, we compared the magnitude of enhancement across the three distraction conditions (ND, ED and DD, Fig. 4). Therefore, a repeated measures ANOVA was conducted with task (WM, passive view), time period (encoding, delay) and Condition (ND, ED, DD) as factors.

There was a significant interaction between time period and task (F(1,26) = 45.36, p < 0.001) as the enhancement was specific to the encoding period for each condition, but not seen for the delay period (main effect of task for the encoding period: F(1,26) = 64.27, p < 0.001; main effect of task for the delay period: F(1,26) = 0.06, p = 0.806). This enhancement did not significantly differ between conditions (there was no significant interaction between task, time and condition: F(2,52) = 0.32, p = 0.726, and no significant interaction between time and condition: F(2,52) = 0.18, p = 0.835). There was significant enhancement for each condition during the encoding period (ND: t(26) = 4.37, p < 0.001, ED: t(26) = 3.71, p < 0.001, DD: t(26) = 2.97, p = 0.006), but not the delay period ND: t(26) = 0.41, p = 0.684, ED: t(26) = − 1.25, p = 0.221, DD: t(26) = 0.08, p = 0.940). Therefore, the enhancement we identified (i.e., greater activity when remembering compared to passively viewing) did not significantly differ across the three distraction conditions, and was confined to the encoding period. This indicates that enhancement was not affected by the inclusion of distractors, or the type of distraction (ED or DD), again failing to support a biased-competition account of distractor resistance.

### Activity associated with the presence or absence of distractors

**Figure 5:**
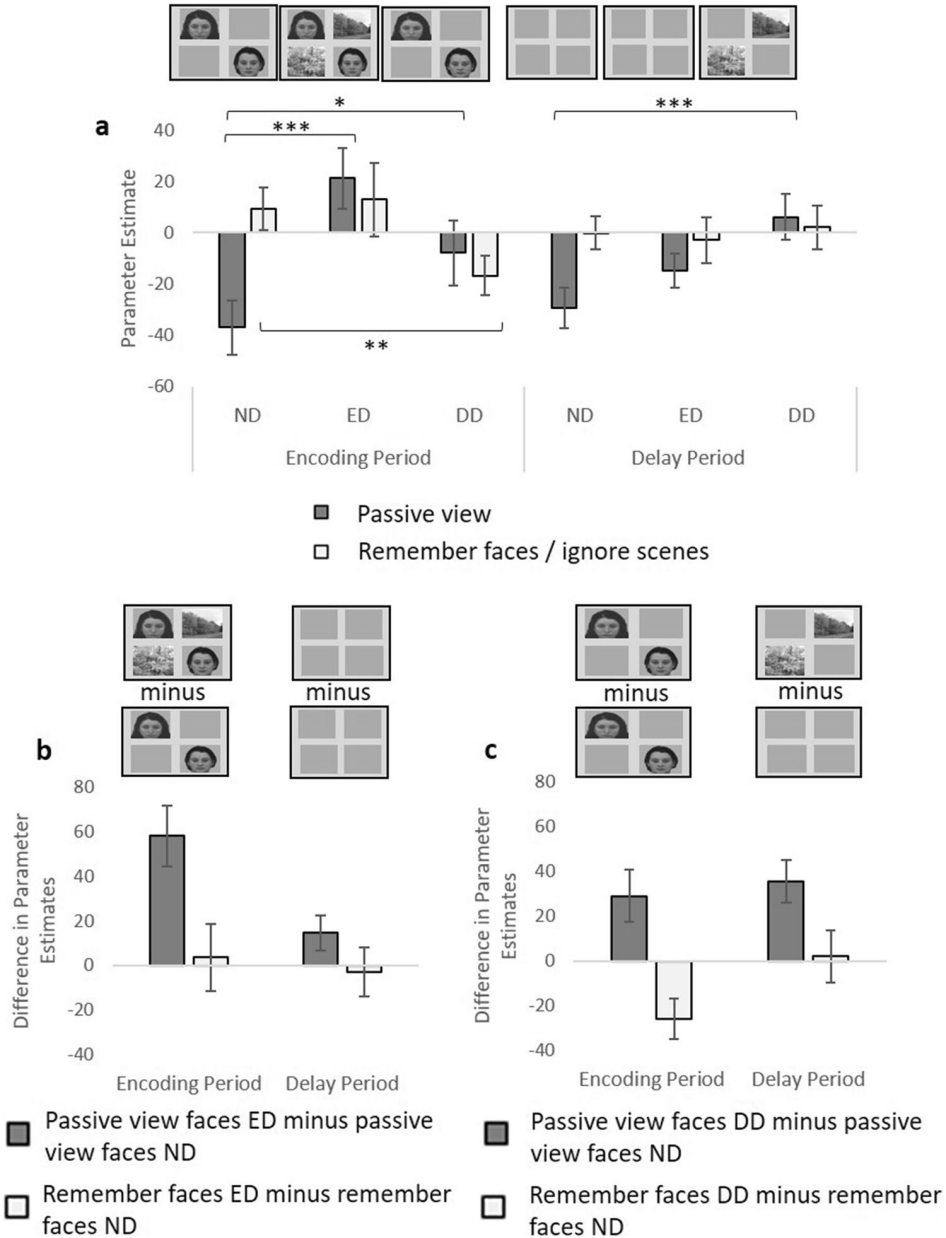
LPPA activity associated with ignoring scenes during the ND, ED and DD conditions, compared to the stimulus-matched passive view conditions. (**a**) LPPA activity when faces should be remembered and scenes (when displayed) should be ignored, for the ND, ED and DD conditions, and also activity for the stimulus-matched passive view task conditions, for both time periods. *p < 0.05, **p < 0.01, ***p < 0.001. (**b**) For illustration we subtracted LPPA activity for the ND condition when faces were shown from LPPA activity for the ED condition during the passive view task, and subtracted LPPA activity for ND condition when faces were to be remembered from LPPA activity for the ED condition when faces should be remembered and scenes ignored. This difference measure represents the extent to which LPPA activity increases when scenes are added to the display during the encoding period under the different task conditions (remember/ ignore versus passive view). The error bars indicate s.e.m. (**c**) Similarly, we subtracted LPPA activity for the ND condition in which faces were displayed and should be passively viewed from LPPA activity for the DD condition in which faces were displayed first, followed by scenes and all stimuli should be passively viewed. We also subtracted LPPA activity for the ND condition in which faces were displayed and should be remembered from LPPA activity for the DD condition in which faces were displayed first and should be remembered and were followed by scenes which should be ignored. This measure represents the extent to which LPPA activity increases when scenes are added to the delay period under the different task conditions (remember/ignore versus passive view). The error bars indicate s.e.m. *p < 0.05, **p < 0.01 and ***p < 0.001.

We next compared LPPA activity when scenes were task-irrelevant stimuli (distractors) across the ND and ED conditions (Fig. 5). Although we did not observe suppression (reduced activity associated with ignoring scenes versus passively viewing the same stimuli) during ED or DD, we sought to discover whether activity associated with presenting distractors (i.e., ED > ND and DD > ND) varied between the passive view and the WM tasks. It is well known that presenting scene stimuli produces a reliable increase in PPA activity during passive view conditions^13^. Should this increase be attenuated when scene stimuli are distractors that should be ignored, this attenuation may represent a mechanism for input-gating.

We first investigated ED, and performed a repeated measures ANOVA with task (passive view, WM), time period (encoding, delay), and condition (ND, ED) as factors. There was a significant interaction between time period, condition and task (F(1,26) = 4.58, p = 0.042). Considering the encoding period, there was a significant interaction between condition and task (F(1,26) = 6.53, p = 0.017). This was driven by significantly greater LPPA activity for the ED condition (when faces and scenes were shown) compared to the ND condition (when only faces were shown) during the passive view task (t(26) = − 4.32, p < 0.001), but no significant difference between these two conditions when faces should be remembered and scenes (when displayed) should be ignored (t(26) = − 0.25, p = 0.807). Therefore, adding scene stimuli to the display of face stimuli significantly increased LPPA activity only during the passive view task, and not when the face stimuli are targets to be remembered and the scene stimuli are distractors to be ignored. In other words, during the WM task, LPPA activity did not significantly change, regardless of the presence or absence of scene distractors, thereby suggesting a mechanism for input gating, or demonstrating a result of such a mechanism.

For the delay period, there was no significant interaction between condition and task (F(1,26) = 1.70, p = 0.204), and no significant main effect of condition (F(1,26) = 0.78, p = 0.385), but there was greater activity for the remember task compared to the passive view task (a significant main effect of task, F(1,26) = 5.04, p = 0.034). Therefore, the significant increase in LPPA activity associated with adding scenes to the display during the encoding period during the passive view task, which was attenuated when those scenes should be ignored (ED), was specific to the encoding period.

We then compared activity in LPPA when scenes were distractors, across the ND and DD conditions (Fig. 5). There was no significant interaction between time period, condition and task (F(1,26) = 2.22, p = 0.149). The encoding period showed a significant increase in activity when a delay distraction was anticipated (t(26) = − 2.49, p = 0.020) compared to the ND condition for the passive view task, but a significant decrease in activity when DD was anticipated for the remember/ignore task (t(26) = 2.85, p = 0.008) compared to the ND condition (condition*task interaction: F(1,26) = 14.28, p <0.001). Similarly, during the delay period there was again a significant interaction between condition and task (F(1,26) = 4.65, p = 0.040), which was this time driven by a significant increase between ND and DD conditions for the passive view task (t(26) = 3.76, p < 0.001) but no significant difference between ND and DD conditions for the remember/ignore task (t(26) = 0.19, p = 0.853). Therefore, as we saw for ED, for DD there was also a significant increase in LPPA activity associated with scene stimuli being displayed, which was not seen when the same scene stimuli should be ignored, but unlike ED, for DD this was observed for both encoding and delay periods.

### Activity associated with processing different distractor types

Finally, we directly compared LPPA activity when scenes should be ignored during the different types of distraction conditions (ED and DD), at the point at which distractors were displayed (the encoding period for ED and the delay period for DD; Fig. 5). As reported above, for both types of distraction there was significantly greater activity for the distraction condition compared to the ND condition for the passive view task but not the remember/ignore task, at the point at which the distractors were displayed, and this did not significantly differ by distraction type (there was no significant interaction between time period, condition (with or without distraction) and task (F(1,26) = 0.71, p = 0.406).

### Eye-closure experiment

The mean duration of eye closures during the 3 s delay period were extremely short duration when remembering faces (127.049 ms, SD = 0.675 ms) and when remembering scenes (131.806 ms, SD = 36.800 ms). Furthermore, the likelihood of eye-closure and the duration of eye-closure did not differ between situations where distractor stimuli were present or absent (present: remembering faces: F(1.205,27.720) = 1.09, p = 0.346, Huynh–Feldt correction; remembering scenes: F(2,46) = 0.34, p = 0.714; absent: remembering faces: F(1.433,32.963) = 1.28, p = 0.282, Huynh–Feldt correction; remembering scenes: F(1.535,35.311 = 0.84, p = 0.414) respectively).

## Discussion

In line with previous studies^14^, which used different WM paradigms, for spatially distinct and anticipated distractors, presented either during WM encoding or maintenance, we observed significantly greater activity in sensory specific regions when their corresponding stimuli were to be remembered versus ignored. This was specific to the encoding period and was driven by enhancement (greater activity when remembering compared to passive view), which did not differ between distractor-present and distractor-absent conditions, or with the type of distraction (encoding vs delay). Importantly, in contrast to previous studies, we did not observe any evidence of suppression (reduction in activity associated with ignoring distractors compared to stimulus-matched passive view conditions). Together, the observation of enhancement, but not suppression, of stimulus specific cortical activity supports an input gating model of distractor processing. Moreover, we observed that adding stimuli to the display significantly increased activity associated with those additional stimuli only for passive view task conditions, not when those stimuli should be ignored in the context of a WM task. This suggests an alternative mechanism of distractor-resistance, more aligned to an input-gating account^9,10^. According to such an account, distractors are blocked from entering WM, and their sensory-specific brain activity need not be reduced.

For both types of distraction (ED and DD), and also the No Distraction condition, we observed significant enhancement in LPPA (greater activity when scenes should be remembered compared to passive view), which was confined to the encoding period. There was no evidence of enhancement during the delay period (although it is not clear whether a post-stimulus undershoot, that did not significantly differ between conditions, may have complicated our ability to measure enhancement or suppression during the delay period; see Fig. 2a). Furthermore, there was no significant difference in enhancement between conditions. Research using transcranial magnetic stimulation (TMS) has provided causal evidence that top-down control signals from the DorsoLateral Prefrontal Cortex (DLPFC) may increase activity in stimulus specific cortical regions related to maintained WM targets when delay period distraction is present^32^. This suggests that activity in target-relevant (stimulus specific) regions may be increased to combat distraction. Yet, our results only exhibited enhancement during the encoding period, not the delay period. This discrepancy may be due to the application of TMS to perturb top-down signals. In that study, they did not reveal whether in the absence of TMS such top-down control signals would elevate target activity for distractor-present trials above that of distractor-absent trials, or whether they serve to maintain equivalent levels of target activity when distractors are added. Our finding of equivalent enhancement (compared to passive view) supports an account in which activity in target-relevant regions is not affected by the presence or nature (ED or DD) of distractors.

Instead of observing reduced activity in LPPA when participants were asked to ignore scenes compared to the stimulus-matched passive view task (i.e., suppression), we observed only equivalent activity between these two tasks. By comparing these tasks across the distractor and no distractor conditions, we were able to observe a different effect. For ED, we observed an expected significant increase in LPPA activity when scene stimuli were added to the display during the passive view task. This is in line with previous research demonstrating that increases in activity in PPA/FFA when scenes/faces are respectively displayed are robust^11–13^. Yet, no significant increase in LPPA activity was observed when the same scene stimuli were added during the WM task, and should be ignored. This same effect was also observed for DD. We propose that this attenuation of that PPA increase under the WM task conditions, when those scene stimuli were to be ignored, represents a potential mechanism of distractor resistance. This is consistent with early selection models of attention^33^ and input-gating accounts^9,10^. Together, these results suggest that input gating may serve to block distraction from WM by stabilizing cortical activity in task-irrelevant (distractor sensitive) cortical regions. This occurs to such an extent that activity in distractor sensitive regions does not differ based on the presence or absence of a distractor.

Although we cannot rule out the possibility of a brief period of suppression that could not be observed with our fMRI procedure, the absence of suppression is in sharp contrast to previous fMRI findings^14–17,22^ and may be explained by differences between paradigms. There are key differences between our WM paradigm and those that have shown suppression. For example, Gazzaley et al.^14^ and Rissman et al.^17^ measured BOLD response averaged across an encoding period that included sequentially presented images of faces and scenes, presented in a random order, such that when an image was displayed, its task-relevance must be assessed and the whole display could then be encoded into memory or ignored accordingly. Chadick and Gazzaley^15^ used sequentially presented superimposed images of faces and scenes, presumably requiring a process of perceptual switching to extract the task-relevant stimulus. Although Haeger et al.^22^ included ND conditions, their approach was to use retro-cues post-encoding to indicate whether a subset of stimuli held in memory were to be subsequently treated as distractors and forgotten. Activity was averaged over stimuli and visual cue presentation and the subsequent maintenance period. With our paradigm, target stimuli were presented simultaneously, but were spatially separate, allowing us to interrogate any distinction between ED and DD. Participants did not need to engage in perceptual switching or assess the relevance of the whole display as they knew the task relevance of upcoming stimuli in advance (for the remember/ignore task there were always two stimuli to remember in the ND condition, two stimuli to remember with two stimuli to ignore during ED, and two stimuli to remember followed by two stimuli to ignore in the DD condition). The targets and distractors were also spatially distinct. It is therefore possible that the suppression reported previously represents a wide-spread reduction of activity associated with ignoring the whole display, or of items that span the whole display, when these are deemed to be task-irrelevant, and that our paradigm requires a more spatially-selective approach.

Alternatively, the absence of significant suppression (ignore < passive view) may be indicative of failed suppression, the distractors not being distracting enough to require suppression, or participants successfully remembering both targets and distractors. However in each of these cases we would have expected to see a rise in distractor-associated activity when the distractors were presented during the WM task, in line with the rise in scene-related activity we observed when scenes were added to the display during the passive view task (Fig. 5), and when participants were asked to remember scenes instead of passively view them (Figs. 2, 3, 4). A rise in scene-associated activity was only seen for the passive view task, not for conditions in which faces should be remembered and scenes ignored (Fig. 5). It therefore seems unlikely that the absence of suppression was due to a failure to ignore the scene distractors.

Furthermore, when remembering scenes, working memory capacity was significantly *higher* for the ED condition compared to the ND condition. The reason for this is not clear. One possibility is that participants may have been less likely to disengage from the more demanding task of remembering some stimuli while ignoring others, and fewer lapses of sustained attention was responsible to superior performance in this condition^34,35^. Importantly, this behavioural result is also inconsistent with an account in which participants failed to effectively ignore the distraction.

It is also important to note that for both time periods, there was a significant increase in LPPA activity when *faces* should be remembered compared to passive view, despite the absence of any scene stimuli within the display (the ND condition). This highlights that, in the absence of overt distraction, top-down modulation is not exclusive to regions associated with target stimuli (PPA when remembering scenes and FFA when remembering faces), but indicates a more wide-spread boost in activity when stimuli should be remembered. Importantly this was not seen during the distractor conditions (ED or DD). As expected, LPPA activity was higher when scenes were added to the screen during passive view, but there was no further boost to this activity when the face stimuli should be remembered compared to passive view. Further research is needed to interrogate the extent to which modulation of this cross-over effect (i.e. enhancement of PPA activity when remembering faces) may contribute to mechanisms supporting distractor resistance. Further work is also needed to interrogate the exact mechanisms by which WM representations, which may be “activity silent”^36^ can be protected from distractor interference.

In summary, our findings demonstrate enhancement of sensory-specific activity that does not specifically contribute to distraction resistance. Furthermore, suppression of activity in sensory-specific cortex (ignoring distractors compared to passive view) was not observed, providing evidence against the biased-competition accounts of distractor resistance. Instead, results support a mechanism of input gating by which sensory-specific activity is stabilized, such that activity does not differ in the presence or absence of distraction.

## Supplementary Information

Supplementary Information.

## Data Availability

The datasets generated and analysed during the current study are available in the OSF repository, https://osf.io/2c3rq/. https://doi.org/10.17605/OSF.IO/2C3RQ.

## References

[1] Vogel EK, McCollough AW, Machizawa MG (1998). Neural measures reveal individual differences in controlling access to working memory. Nature.

[2] McNab F, Klingberg T (2008). Prefrontal cortex and basal ganglia control access to working memory. Nat. Neurosci..

[3] Hasher L, Zacks RT, Bower GH (1988). Working memory, comprehension, and aging: A review and anew view. The Psychology of Learning and Motivation.

[4] Lee F-Y, Cowan N, Vogel EK, Rolan T, Valle-Inclán F, Hackley SA (2010). Visual working memory deficits in patients with Parkinson’s disease are due to both reduced storage capacity and impaired ability to filter out irrelevant information. Brain.

[5] Lorenc ES, Mallett R, Lewis-Peacock JA (2021). Distraction in visual working memory: Resistance if not futile. Trends Cogn. Sci..

[6] Feldmann-Wüstefeld T, Vogel EK (2019). Neural evidence for the contribution of active suppression during working memory filtering. Cereb. Cortex.

[7] Allon AS, Luria R (2018). Filtering performance in visual working memory is improved by reducing early spatial attention to the distractors. Psychophysiology.

[8] Liesefeld HR, Liesefeld AM, Sauseng P, Jacob SN, Müller HJ (2020). How visual working memory handles distraction: Cognitive mechanisms and electrophysiological correlates. Vis. Cogn..

[9] Chatham CH, Badre D (2015). Multiple gates on working memory. Curr. Opin. Behav. Sci..

[10] O’Reilly RC, Frank MJ (2006). Making working memory work: A computational model of learning in the prefrontal cortex and basal ganglia. Neural Comput..

[11] Kanwisher N, McDermott J, Chun MM (1997). The fusiform face area: A module in human extrastriate cortex specialized for face perception. J. Neurosci..

[12] Puce A, Allison T, Gore JC, McCarthy G (1995). Face-sensitive regions in human extrastriate cortex studies by functional MRI. J. Neurophysiol..

[13] Epstein R, Kanwisher N (1998). A cortical representation of the local visual environment. Nature.

[14] Gazzaley A, Cooney JW, McEvoy K, Knight RT, D’Esposito M (2005). Top-down enhancement and suppression of the magnitude and speed of neural activity. J. Cogn. Neuro.

[15] Chadick JZ, Gazzaley A (2011). Differential coupling of visual cortex with default network or frontal-parietal network based on goals. Nat. Neurosci..

[16] Rutman AM, Clapp WC, Chadick JA, Gazzaley A (2010). Early top-down control of visual processing predicts working memory performance. J. Cogn. Neurosci..

[17] Rissman J, Gazzaley A, D’Esposito M (2009). The effect of non-visual working memory load on top-down modulation of visual processing. Neuropsychologia.

[18] Gazzaley A, Cooney JW, Rissman J, D’Esposito M (2005). Top-down suppression deficit underlies working memory impairment in normal aging. Nat. Neurosci..

[19] Chadick JZ, Zanto TP, Gazzaley A (2014). Structural and functional differences in prefrontal cortex underlie distractibility and suppression deficits in ageing. Nat. Commun..

[20] Gazzaley A, Clapp W, McEvoy K, Knight R, D’Esposito M (2008). Age-related top-down suppression deficit in the early stages of cortical visual memory processing. Proc. Natl. Acad. Sci..

[21] Zanto TP, Gazzaley A (2009). Neural suppression of irrelevant information underlies optimal working memory performance. J. Neurosci..

[22] Haeger A, Lee H, Fell J, Axmacher N (2015). Selective processing of buildings and faces during working memory: the role of the ventral striatum. Eur. J. Neurosci..

[23] McNab F, Dolan RJ (2014). Dissociating distractor-filtering at encoding and during maintenance. JEP HPP.

[24] McNab F, Zeidman P, Rutledge RB, Smittenaar P, Brown HR, Adams RA, Dolan RJ (2015). Age-related changes in working memory and the ability to ignore distraction. PNAS.

[25] Smith SM (2002). Fast robust automated brain extraction. Hum. Brain Mapp..

[26] Jenkinson M, Bannister P, Brady M, Smith S (2002). Improved optimization for the robust and accurate linear registration and motion correction of brain images. Neuroimage.

[27] Jenkinson M, Smith S (2002). A global optimisation method for robust affine registration of brain images. Med. Image Anal..

[28] Andersson, J. L. R., Jenkinson, M. & Smith, S. Non-linear registration aka Spatial normalisation FMRIB technical report TR07JA2. In *FMRIB Analysis Group of the University of Oxford*, 1–22 (2007).

[29] Andersson, J. L. R., Jenkinson, M. & Smith, S. Non-linear optimisation. In *FMRIB Technical Report TR07JA1* (2007).

[30] Woolrich MW, Ripley BD, Brady M, Smith SM (2001). Temporal autocorrelation in univariate linear modeling of FMRI data. Neuroimage.

[31] Cowan N (2001). The magical number 4 in short-term memory: A reconsideration of mental storage capacity. Behav. Brain Sci..

[32] Feredoes E, Heinen K, Weiskopf N, Ruff C, Driver J (2011). Causal evidence for frontal involvement in memory target maintenance by posterior brain areas during distractor interference of visual working memory. Proc. Natl. Acad. Sci..

[33] Broadbent DE (1958). Perception and Communication.

[34] Smallwood J, Davies JB, Heim D, Finnigan F, Sudbury M, O’Connor R, Obonsawin M (2004). Subjective experience and the attentional lapse: Task engagement and disengagement during sustained attention. Conscious. Cogn..

[35] de Bettencourt MT, Norman KA, Turk-Browne NB (2017). Forgetting from lapses of sustained attention. Psychon. Bull. Rev..

[36] Stokes MG (2015). “Activity-silent” working memory in prefrontal cortex: A dynamic coding framework. Trends Cogn. Sci..

