# Supplementary material for: Stimulus specific cortical activity associated with ignoring distraction during working memory encoding and maintenance
Source: Sci Rep. 2023 Jun 2;13:8952. doi: 10.1038/s41598-023-34967-2 (PMC10238503; doi:10.1038/s41598-023-34967-2)
Supplement: Supplementary file 1 — Supplementary Information. [file 41598_2023_34967_MOESM1_ESM.docx]

Supplementary Material

Here we report the results of ANOVAs for the four ROIs, including the three not reported in the manuscript. We compared ROIs by including a factor for ROI in our ANOVAs, but also report the results of the separate ANOVAs for each ROI for completeness, irrespective of whether there was a significant interaction involving ROI.

We also include analysis to show how activity was modulated by whether the face / scene stimuli should be remembered or ignored. With repeated measures ANOVAs and follow-up paired-tests we examined the effects of time period (encoding period or delay period) and task (Remember or Ignore). We first considered ED and then repeated this approach for DD. We did not include ED/DD as a factor within the ANOVA given that they involve distractor presentation during different time periods. For a direct comparison between ED and DD we later compared the encoding period for ED with the delay period for DD (see below).

Modulation of BOLD response with task – ED

We first considered ED and the results are shown in Figures S1 and S2. As anticipated there was significantly greater activity associated with remembering compared to ignoring (a significant main effect of task: F(1,26)=26.56, p<0.001), which significantly differed by ROI and time period (significant ROI*time period*task interaction: F(1.65,43.00)=4.43, p=0.024; Greenhouse-Geisser as W(5)=0.30, p<0.001). With separate ANOVAs for each ROI we found that for all ROIs except LFFA there was a significant interaction between time period and task (RPPA: F(1,26)=31.76, p<0.001, Figure S1a, LPPA: F(1,26)=19.35, p<0.001, Figure S1c, RFFA: F(1,26)=8.63, p=0.007, Figure S2a, LFFA: F(1,26)=2.50, p=0.126, Figure S2c). During the encoding period there was significantly greater activity for remembering compared to ignoring (RPPA: t(26)=3.39, p=0.002, Figure S1a, LPPA: t(26)=3.27, p=0.003, Figure S1c, RFFA: t(26)=2.80, p=0.010, Figure S2a). During the delay period, there was significantly *reduced* activity when remembering compared to ignoring for RPPA (t(26)=-4.39, p<0.001, Figure S1a) and for LPPA and RFFA there was no significant difference between tasks (LPPA: t(26)=-1.73, p=0.095, Figure S1c; RFFA: t(26)=-0.05, p=0.959, Figure S2a). For LFFA the interaction between time period and task failed to reach significance (F(1,26)=2.50, p=0.126) and activity did not significantly differ between the two tasks (F(1,26)=1.21, p=0.281) (Figure S2c).

*
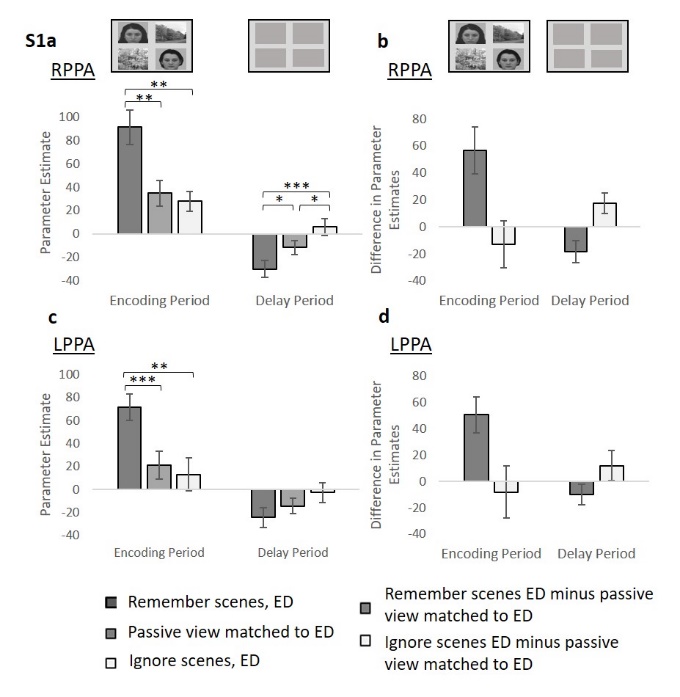
*

*Figure S1, PPA activity associated with remembering and ignoring scenes during the ED condition, compared to the stimulus-matched PV condition. a) RPPA activity and c) LPPA activity for the ED condition when the task was to remember scenes/ignore faces, passively view scenes and faces, and remember faces/ignore scenes, for the encoding and delay period. The stimuli displayed for each time period are shown at the top of the figure. For illustration, we subtracted passive view activity from each of the other two task conditions, to show the level of enhancement and suppression for RPPA (b) and LPPA (d). The error bars indicate s.e.m. and for (a) and (c), * p < 0.05, ** p < 0.01, *** p < 0.001.*


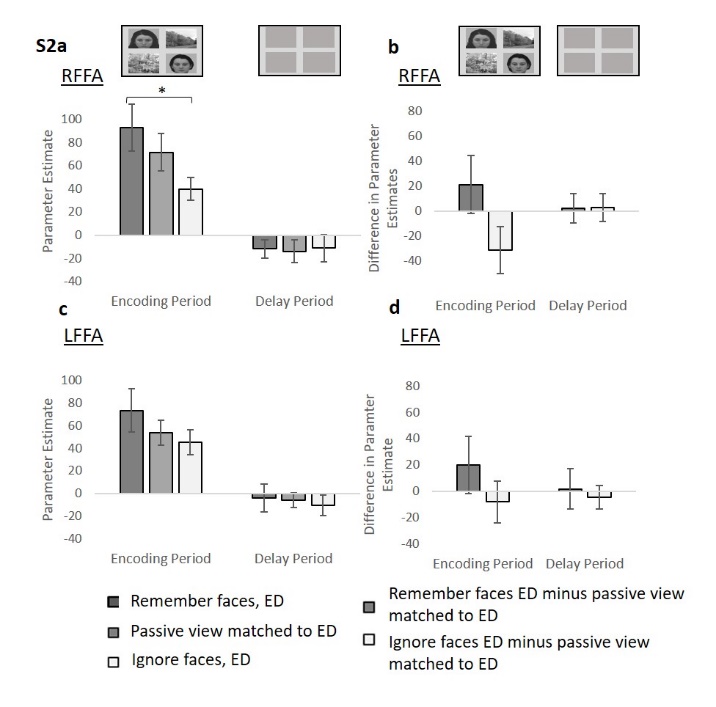


*Figure S2, FFA activity associated with remembering and ignoring faces during the ED condition, compared to the stimulus-matched PV condition. a) RFFA activity and c) LFFA activity for the ED condition when the task was to remember faces/ignore scenes, passively view faces and scenes, and remember scenes/ignore faces, for the encoding and delay period. The stimuli displayed for each time period are shown at the top of the figure. For illustration, we subtracted passive view activity from each of the other two task conditions, to show the level of enhancement and suppression for RFFA (b) and LFFA (d). The error bars indicate s.e.m. and for (a) and (c), * p < 0.05.*

Modulation of BOLD response with task – DD

The results are shown in Figures S3 and S4. As with ED, there was significant modulation of activity with task (remember versus ignore; a significant main effect of task: F(1,26)=17.85, p<0.001), but there was also a significant interaction between ROI, time period and task (F(1.45,37.56)=4.73, p=0.024; Greenhouse-Geisser as W(5)=0.15, p<0.001).

For all ROIs there was a significant interaction between time period and task (RPPA: F(1,26)=55.00, p<0.001, Figure S3a, LPPA: F(1,26)=56.55, p<0.001, Figure S3c, RFFA: F(1,26)=16.49, p<0.001, Figure S4a, LFFA: F(1,26)=8.33, p=0.008, Figure S4c). Paired t-tests revealed that during the encoding period there was significantly greater activity when remembering compared to ignoring for PPA but not FFA ROIs (RPPA: t(26)=4.19, p<0.001, Figure S3a, LPPA: t(26)=4.66, p<0.001, Figure S3c, RFFA: t(26)=1.94, p=0.063, Figure S4a, LFFA: t(26)=1.16, p=0.256, Figure S4c). For the delay period there was *reduced* activity for the remember task compared to the ignore task for the FFA ROIs (RFFA: t(26)=-4.13, p<0.001, Figure S4a, LFFA: t(26)=-2.28, p=0.031, Figure S4c) but no significant difference for the PPA ROIs (RPPA: t(26)=-1.55, p=0.134, Figure S3a, LPPA: t(26)=-0.98, p=0.335, Figure S3c).


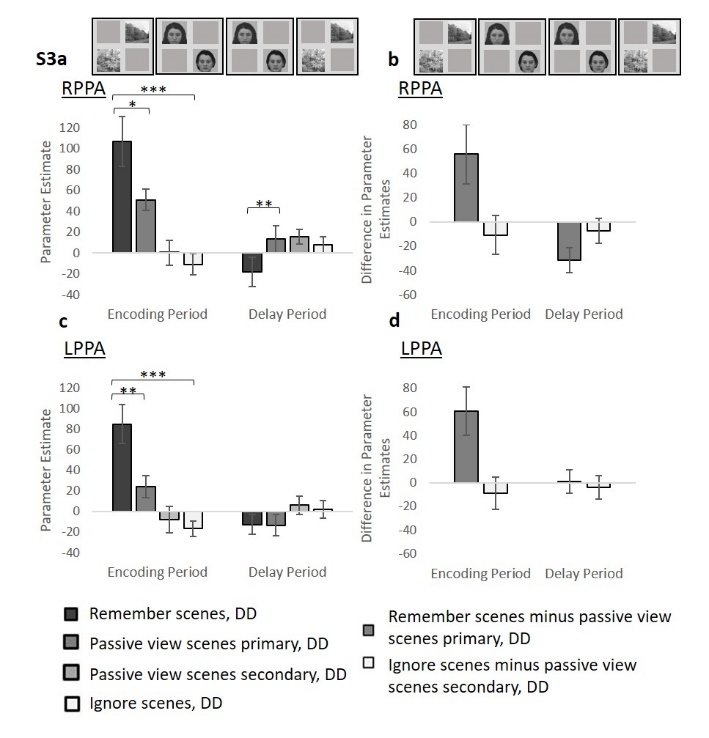


*Figure S3, PPA activity associated with remembering and ignoring scenes during the DD condition, compared to the stimulus-matched PV conditions. a) RPPA activity and c) LPPA activity for the DD condition when the task was to remember scenes during the encoding period and ignore faces during the delay period, passively view scenes during the “encoding period” and then passively view faces during the “delay period”), passively view faces during the “encoding period” and then passively view scenes during the “delay period”, and remember faces during the encoding period and ignore scenes during the delay period. The stimuli displayed for each time period are shown at the top of the figure. For illustration, we subtracted passive view activity from its stimulus-matched remember/ignore task condition, to show the level of enhancement and suppression for RPPA (b) and LPPA (d). The error bars indicate s.e.m. and for (a) and (c), * p < 0.05, ** p < 0.01, *** p < 0.001.*


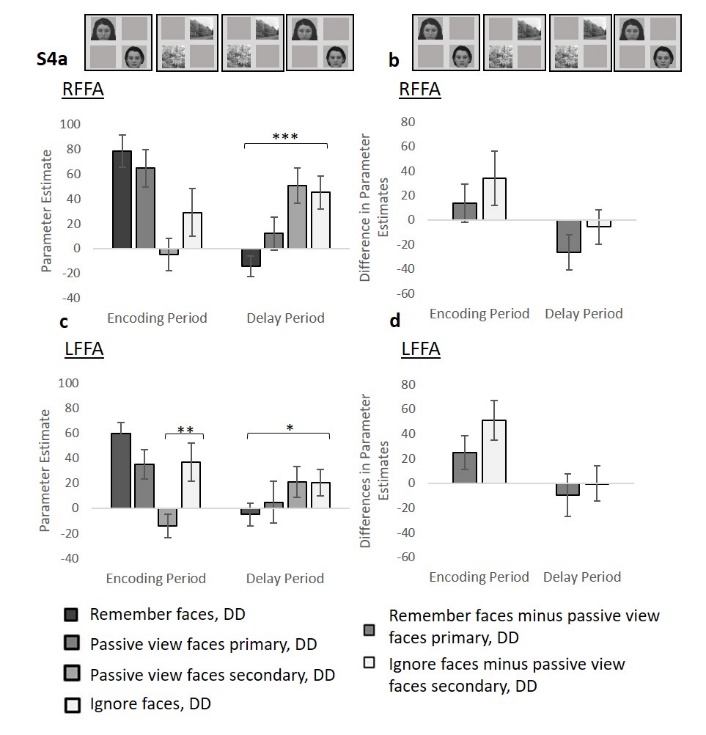


*Figure S4, FFA activity associated with remembering and ignoring faces during the DD condition, compared to the stimulus-matched PV conditions. FFA activity associated with remembering, passively viewing and ignoring RFFA activity and c) LFFA activity for the DD condition when the task was to remember faces/ignore scenes, passively view faces and then scenes, passively view scenes and then faces, and remember scenes/ignore faces, for the encoding and delay period. The stimuli displayed for each time period are shown at the top of the figure. For illustration, we subtracted passive view activity from its stimulus-matched remember/ignore task condition, to show the level of enhancement and suppression for the RFFA (b) and LFFA (d). The error bars indicate s.e.m. and for (a) and (c), * p < 0.05, ** p < 0.01, *** p < 0.001.*

Remember compared to the passive view baseline – ED

Having identified greater activity for remembering compared to ignoring (ED) during the encoding period for each ROI except LFFA, we next investigated the extent to which this modulation was driven by enhancement of activity when remembering stimuli compared to stimulus-matched PV conditions, as shown in Figures S1 and S2. There was again a significant interaction between ROI, time and task (F(1.82,47.31)=4.92, p=0.014; Greenhouse-Geisser as W(5)=0.38, p<0.001). For RPPA and LPPA the effect of task significantly differed between the two time periods (RPPA: F(1,26)=16.06, p<0.001, LPPA: F(1,26)=13.88, p<0.001). There was significant enhancement during the encoding period for RPPA (t(26)=3.24, p=0.003, Figure S1a) and LPPA (t(26)=3.71, p<0.001, Figure S1c) but for the delay period there was significantly *lower* activity for the remember compared to the PV task for RPPA (t(26)=-2.27, p=0.031), and no significant difference between tasks for LPPA (t(26)=-1.25, p=0.221). For FFA there was no significant difference between the remember and PV task conditions (main effect of task for RFFA: F(1,26)=0.61, p=0.441, Figure S2a, and for LFFA: F(1,26)=0.38, p=0.542, Figure S2c) and no significant interaction between task and time period (RFFA: F(1,26)=0.77, p=0.388, LFFA: F(1,26)=1.93, p=0.176).

Therefore, for both PPA ROIs, and for the encoding period only, significant enhancement was observed in the ED condition when scene stimuli were to be remembered compared to the stimulus-matched baseline PV.

Ignore compared to the passive view baseline – ED

To investigate suppression during the ED task, again taking the approach of Gazzaley et al [1], we compared activity during ignore (ED) versus stimulus-matched PV conditions. The results are shown in Figures S1 and S2. Across the 4 ROIs there was no main effect of task (F(1,26)=0.20, p=0.655), and no significant interaction between task and ROI (F(2.22,57.7)=0.79, p=0.471; Greenhouse-Geisser as W(5)=0.54, p=0.009), task and time period (F(1,26)=2.75, p=0.110) or between ROI, time and task (F(2.37,61.56)=1.80, p=0.167; Greenhouse-Geisser as W(5)=0.62, p=0.04).

For RPPA (Figure S1a) there was a significant interaction between time period and task (F(1,26)=4.34, p=0.047), but this was driven by a significant task difference in the delay period (t(26)=-2.30, p=0.030) but not the encoding period (t(26)=0.74, p=0.465) and it was due to *greater* activity for the ignore task compared to PV. For LPPA (Figure S1c), RFFA (Figure S2a) and LFFA (Figure S2c) there was no significant task difference (LPPA: F(1,26)=0.02, p=0.899, RFFA: F(1,26)=1.38, p=0.250, LFFA: F(1,26)=0.39, p=0.540) and no significant task*time period interaction (LPPA: F(1,26)=1.63, p=0.213, RFFA: F(1,26)=3.43, p=0.075, LFFA: F(1,26)=0.05, p=0.825). Therefore, no ROI showed significantly lower activity for the ignore ED task compared to the stimulus-matched PV task.

Although the ANOVAs for RFFA had not provided any evidence of enhancement or suppression compared to the PV task, as we had observed a significant difference between the remember versus ignore task in the encoding period for RFFA (Figure S2a), we further probed this difference with paired samples t-tests. There was no significant difference between the remember and PV task (t(26)=0.91, p=0.372) and no significant difference between activity associated with the ignore task compared to PV (t(26)=1.69, p=0.104.

We also repeated the analysis with ROI values averaged across the two hemispheres to ensure we were not missing significant effects by considering each ROI separately. For both PPA and FFA there was no significant main effect of task (PPA: F(1,26)=0.03, p=0.864; FFA: F(1,26)=1.16, p=0.292) and no significant interaction between time and task (PPA: F(1,26)=3.14, p=0.088; FFA: F(1,26)=1.52, p=0.229). There was no significant task difference (PPA: encoding period: t(26)=0.59, p=0.558; delay period (t(26)=-1.70, p=0.101; FFA: encoding period: t(26)=1.27, p=0.215; delay period: t(26)=0.13, p=0.897), and delay period PPA activity was again numerically *greater* for the ignore task compared to PV.

Remember compared to the passive view baseline – DD

To investigate enhancement for DD, we compared the remember condition with its stimulus-matched PV condition. There was no significant interaction between task and ROI (F(1.61,49.92)=1.55, p=0.225; Greenhouse-Geisser as W(5)=0.19, p<0.001) or between task, time period and ROI (F(1.35,35.21)=2.04, p=0.158; Greenhouse-Geisser as W(5)=0.11, p<0.001). Separate ANOVAs for each ROI revealed a significant interaction between time period and task for all ROIs except LFFA (RPPA: F(1,26)=14.12, p<0.001, Figure S3a, LPPA: F(1,26)=9.85, p=0.004, Figure S3c, RFFA: F(1,26)=7.60, p=0.011, Figure S4a, LFFA: F(1,26)=3.83, p=0.061, Figure S4c). Paired t-tests identified significant enhancement during the encoding period for RPPA (t(26)=2.29, p=0.030 and LPPA (t(26)=2.97, p=0.006) but not RFFA (t(26)=0.89, p=0.382) or LFFA (t(26)=1.80, p=0.084). During the delay period there was only a difference between remember and PV for RPPA, and this was associated with *lower* activity for remember versus PV (t(26)=-3.08, p=0.005). For all other ROIs, the paired t-tests between the remember and PV tasks gave p > 0.077.

Therefore, as with ED, for the DD condition, for both PPA ROIs, and for the encoding period only, significant enhancement was observed when scene stimuli were to be remembered compared to the stimulus-matched baseline PV.

Ignore compared to the passive view baseline – DD

To investigate suppression due to DD, we compared ignore (DD) versus PV tasks and the results are shown in Figures S3 and S4. There was a significant interaction between ROI, time period and task (F(1.51,39.35)=3.99, p=0.037; Greenhouse-Geisser as W(5)=0.20, p<0.001). Separate ANOVAs for each ROI revealed a significant interaction between time and task only for LFFA (main effect of task: RPPA: F(1,26)=0.59, p=0.450, Figure S3a, LPPA: F(1,26)=0.34, p=0.567, Figure S3c, RFFA: F(1,26)=0.82, p=0.375, Figure S4a, LFFA: F(1,26)=4.23, p=0.050, Figure S4c, interaction between time period and task: RPPA: F(1,26)=0.08, p=0.780, LPPA: F(1,26)=0.27, p=0.611, RFFA: F(1,26)=4.21, p=0.051), LFFA: F(1,26)=8.74, p=0.007). Paired t-tests showed that the LFFA task effect was driven by significantly *higher* activity for the ignore condition compared to the stimulus-matched PV baseline for the encoding period only (encoding period: t(26)=3.22, p=0.003, delay period: t(26)=-0.03, p=0.978). Therefore, as with ED, for DD there was no evidence of significantly lower activity for the ignore DD task when compared to its stimulus-matched PV task.

We also repeated the analysis with ROI values averaged across the two hemispheres to ensure we were not missing significant effects by considering each ROI separately. For both PPA and FFA there was no significant main effect of task (PPA: F(1,26)=0.58, p=0.453; FFA: F(1,26)=2.69, p=0.113) and for PPA there was no significant interaction between time and task (PPA: F(1,26)=0.19, p=0.669). For FFA there was a significant interaction between time and task (F(1,26)=6.90, p=0.014). There was no significant task difference for PPA (encoding period: t(26)=-0.76, p=0.455; delay period (t(26)=-0.62, p=0.543). For FFA there was a significant task difference for the encoding period (t(26)=2.51, p=0.019), due to *higher* activity when ignoring faces compared to passive view. For the delay period there was no significant FFA task difference (t(26)=-0.24, p=0.810).

Task-relevant activity compared across distraction conditions

Having established significant enhancement in PPA during the encoding period for both ED and DD conditions, we compared remember versus PV activity across the three distraction conditions (ND, ED and DD, Figures S5 and S6). There was a significant interaction between ROI, time period and task (F(1.83,47.55)=6.24, p=0.005; Greenhouse-Geisser as W(5)=0.31, p<0.001), but no significant interaction involving task and condition (p>0.589 for all interactions involving task and condition).


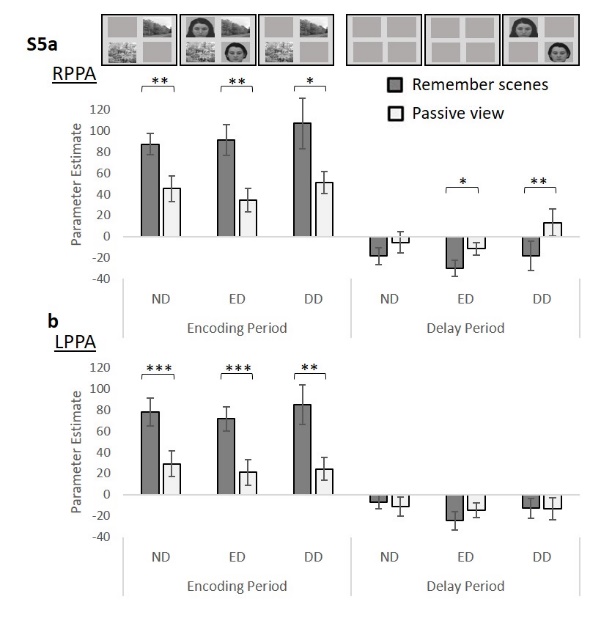


*Figure S5, PPA activity associated with remembering scenes during the ND, ED and DD conditions, compared to the stimulus-matched PV conditions. a) RPPA and b) LPPA activity when scenes should be remembered and faces should be ignored for the ND, ED and DD conditions, and also for the stimulus-matched passive view task, for both time periods. The error bars indicate s.e.m. and for (a) and (c), * p < 0.05, ** p < 0.01, *** p < 0.001.*


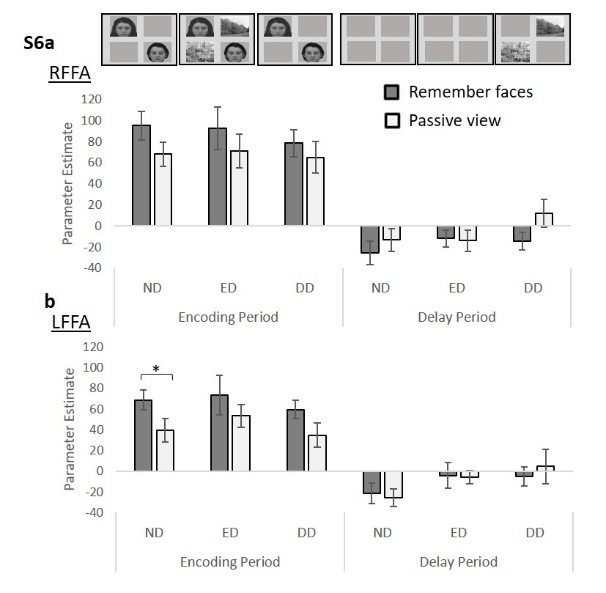


*Figure S6, FFA activity associated with remembering faces during the ND, ED and DD conditions, compared to the stimulus-matched PV conditions. a) RFFA and b) LFFA activity when faces should be remembered and scenes should be ignored for the ND, ED and DD conditions, and also for the stimulus-matched passive view task, for both time periods. The error bars indicate s.e.m. and * p < 0.05.*

Considering each ROI separately, for RPPA and LPPA there was a significant interaction between time period and task (RPPA: F(1,26)=86.80, p<0.001, Figure S5a, LPPA: F(1,26)=45.36, p<0.001, Figure S5c) but no significant effect of condition (p>0.352 for each interaction involving condition as well as the main effect of condition). For the encoding period, there was greater activity for the remember task compared to the PV task (main effect of task: RPPA: F(1,26)=64.27, p<0.001, LPPA: F(1,26)=43.70, p<0.001) and paired t-tests showed significant enhancement for each condition (RPPA: ND: t(26)=3.42, p=0.002, ED: t(26)=3.24, p=0.003, DD: t(26)=2.29, p=0.030, LPPA: ND: t(26)=4.37, p<0.001, ED: t(26)=3.71, p<0.001, DD: t(26)=2.97, p=0.006). For the delay period there was *lower* activity for the remember task compared to the PV task for RPPA (main effect of task: F(1,26)=12.46, p=0.002), which reached significance for ED (t(26)=-2.27, p=0.031) and DD (t(26)=-3.08, p=0.005) but not ND (t(26)=-0.91, p=0.372; although there was no significant interaction between condition and task: F(2,52)=0.67, p=0.515). For LPPA there was no difference between the remember and PV tasks (main effect of task: F(1,26)=0.06, p=0.806) and this did not significantly differ by condition (F(2,52)=0.65, p=0.529).

For RFFA and LFFA there was again a significant interaction between time period and task (RFFA: F(1,26)=7.31, p=0.012, Figure S6a), LFFA: F(1,26)=6.13, p=0.020, Figure S6c), with higher activity for the remember task compared to PV for the encoding period, but lower or similar activity for the remember task compared to PV for the delay period. When considering the two time periods separately, we found significant enhancement for LFFA (a significant main effect of task: F(1,26)=4.50, p=0.044) during the encoding period, but not the delay period (F(1,26)=0.02, p=0.891). For RFFA there was no main effect of task for either time period (encoding period: F(1,26)=2.93, p=0.099, delay period: F(1,26)=2.38, p=0.135). The conditions differed across the two time periods (RFFA: F(2,52)=3.41, p=0.041, LFFA: F(2,52)=4.08, p=0.023). There was no significant difference between conditions (encoding period: RFFA: F(2,52)=0.617, p=0.543, LFFA: F(2,52)=1.57, p=0.218; delay period: LFFA: F(2,52)=2.78, p=0.071 but not RFFA: (F(2,52)=2.35, p=0.105).

Importantly there was no evidence that the effect of task differed by condition (there was no significant interaction involving task and condition, p>0.571). Therefore, the enhancement we identified (greater activity for remember compared to PV task conditions) for PPA but not FFA ROIs, and which was confined to the encoding period, was equivalent for each of the three distraction conditions. It was not affected by the inclusion of distractors, or the type of distraction (ED or DD).

Task-irrelevant activity compared across ND and ED conditions during the encoding period

We next compared activity associated with the task-irrelevant stimuli across the ND and ED conditions (Figures S7 – S10). We had not observed suppression (reduced activity associated with ignoring versus PV) for ED, but sought to discover how activity associated with task-irrelevant stimuli varied between the ND and distraction (ED) conditions when task-relevant stimuli should be remembered and (in the case of ED) task-irrelevant stimuli should be ignored, compared to stimulus-matched PV conditions.

There was a significant interaction between ROI and time period (F(3,78)=7.85, p<0.001) but ROI did not significantly interact with any other variables (p>0.103) .

For all ROIs there was a significant interaction between time period, condition and task (RPPA: F(1,26)=5.24, p=0.030, Figure S7a, LPPA: F(1,26)=4.58, p=0.042, Figure S7b, RFFA: F(1,26)=11.71, p=0.002, Figure S8a, LFFA: F(1,26)=6.15, p=0.020, Figure S8b).

Considering the encoding period, for LPPA, RFFA and LFFA there was a significant interaction between condition and task (LPPA: F(1,26)=6.53, p=0.017, RFFA: F(1,26)=5.73, p=0.024, LFFA: F(1,26)=7.72, p=0.010), which was driven by a significant difference between ND and ED conditions for the PV task (LPPA: t(26)=-4.32, p<0.001, RFFA: t(26)=-3.90, p<0.001, LFFA: t(26)=-5.44, p<0.001) but not the remember/ignore task (LPPA: t(26)=-0.25, p=0.807, RFFA: t(26)=-0.84, p=0.408, LFFA: t(26)=1.71, p=0.099). For RPPA, although the condition * task interaction was not significant (F(1,26)=2.24, p=0.147), the same pattern was observed, with a significant increase in activity when ED were added to the display for the PV task (t(26)=-3.68, p=0.001, and a smaller increase for the remember/ignore task, with this increase not reaching statistical significance (t(26)=-1.87, p=0.073).

For the delay period, and for all ROIs, there was no significant interaction between condition and task (RPPA: F(1,26)=0.38 p=0.545, LPPA: F(1,26)=1.70, p=0.204, RFFA: F(1,26)=0.58, p=0.454, LFFA: F(1,26)=0.40, p=0.531), and no significant main effect of condition (RPPA: F(1,26)=1.29, p=0.266, LPPA: F(1,26)=0.78, p=0.385, RFFA: F(1,26)=0.09, p=0.766, LFFA: F(1,26)=1.75, p=0.197). For LPPA there was greater activity for the remember task compared to the PV task (a significant main effect of task, F(1,26)=5.04, p=0.034), but for the other ROIs there was no significant main effect of task (RPPA: F(1,26)=3.53, p=0.072, RFFA: F(1,26)=0.31, p=0.584, LFFA: F(1,26)<0.01, p=0.996).

Therefore, we consistently observed that the increase in task-irrelevant stimulus activity associated with adding task-irrelevant stimuli to the display during the encoding period was significantly attenuated for the remember/ignore task compared to the PV task, and that this effect was confined to the encoding period.

Task-irrelevant activity compared across ND and DD conditions

We then compared activity associated with the task-irrelevant stimuli across the ND and DD conditions, focusing on the delay period (Figures S7 – S10). There was no significant interaction between ROI, time period, condition and task (F(1.502,39.060)=9.38, p=0.376, Greenhouse-Geisser as W(5)=0.19, p<0.001).


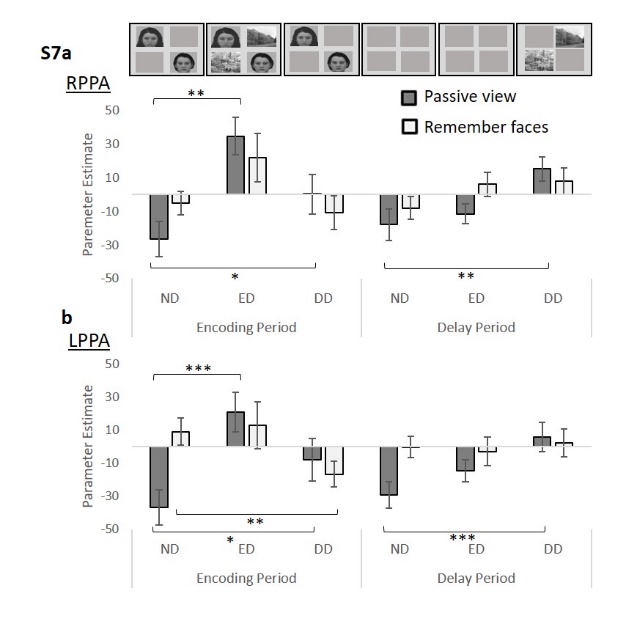


*Figure S7, PPA activity associated with ignoring scenes during the ND, ED and DD conditions, compared to the stimulus-matched PV conditions. a) RPPA and b) LPPA activity when faces should be remembered (and for ED and DD scenes should be ignored) shown for the ND, ED and DD conditions, and also activity for the stimulus-matched passive view task conditions, for both time periods. The error bars indicate s.e.m. and * p < 0.05, ** p < 0.01, *** p < 0.001.*


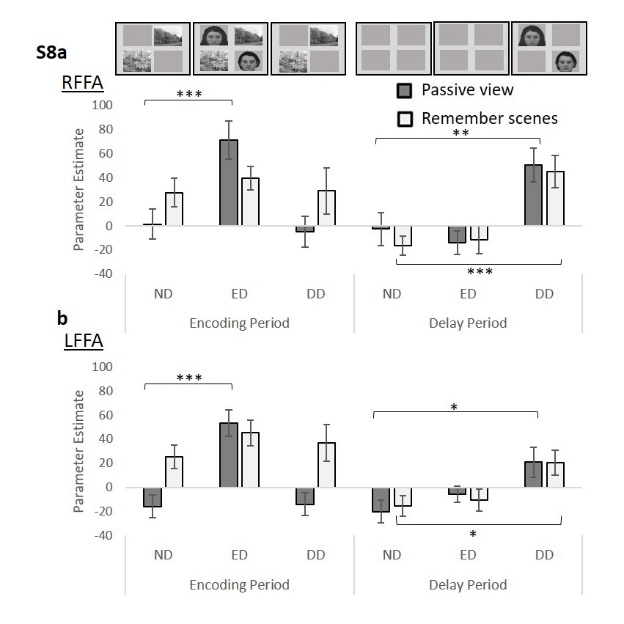


*Figure S8, FFA activity associated with ignoring faces during the ND, ED and DD conditions, compared to the stimulus-matched PV conditions. RFFA and b) LFFA activity when scenes should be remembered (and for ED and DD faces should be ignored) shown for the ND, ED and DD conditions, and also activity for the stimulus-matched passive view task conditions, for both time periods. The error bars indicate s.e.m. and * p < 0.05, *** p < 0.01, *** p < 0.001.*


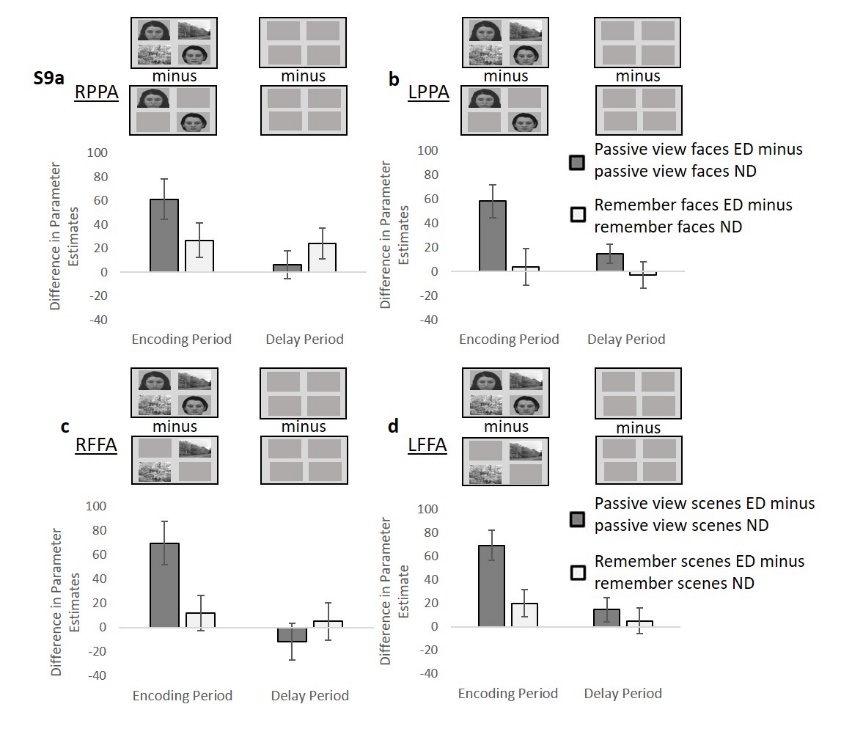


*Figure S9, Changes in activity associated with the inclusion of task-irrelevant during encoding. For illustration we subtracted task-irrelevant stimulus activity for the ND condition from task-irrelevant stimulus activity for the ED condition, for both passive view and working memory tasks, to represent the extent to which this activity increases when ED are added to the display. The figure shows a) RPPA activity and b) LPPA activity when faces were to be remembered and scenes were distractors, as well as c) LFFA activity and d) RFFA activity when scenes were to be remembered and faces were distractors. The error bars indicate s.e.m.*


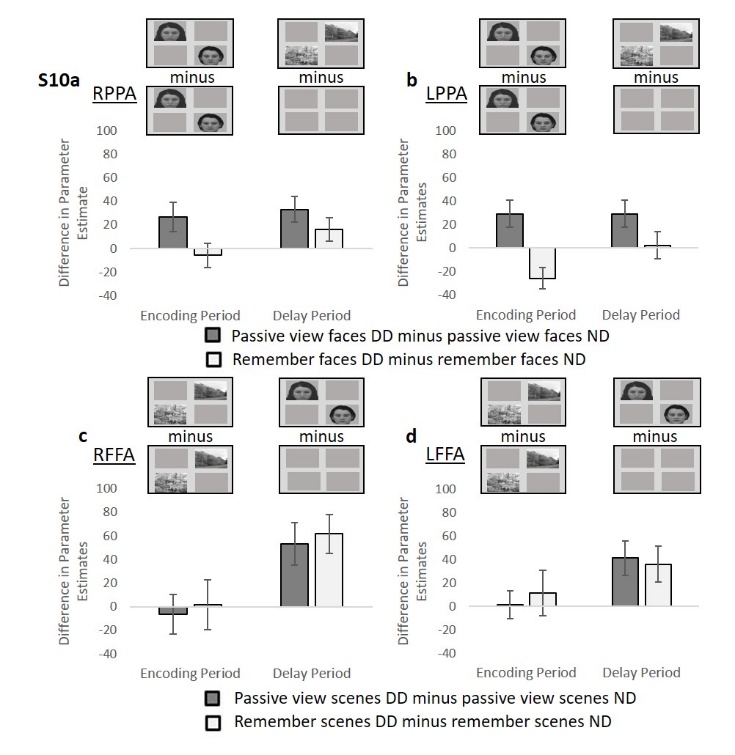


*Figure S10, Changes in activity associated with the inclusion of task-irrelevant stimuli during the delay period. For illustration we subtracted task-irrelevant stimulus activity for the ND condition from task-irrelevant stimulus activity for the DD condition, for both passive view and working memory tasks, to represent the extent to which this activity increases when DD are added to the display. The figure shows a) RPPA activity and b) LPPA activity when faces were to be remembered and scenes were distractors, as well as c) LFFA activity and d) RFFA activity when scenes were to be remembered and faces were distractors. The error bars indicate s.e.m.*

For all ROIs there was no significant interaction between time period, condition and task (RPPA: F(1,26)=0.89, p=0.354, Figure S7a, LPPA:F(1,26)=2.22, p=0.149, Figure S7b, RFFA: F(1,26)<0.01, p=0.976, Figure S8a, LFFA: F(1,26)=0.49, p=0.489, Figure S8b). We report effects of condition and task for each time period.

For LPPA (Figure S7b), the encoding period showed a significant increase in activity when DD was anticipated (t(26)=-2.49, p=0.020) compared to the ND condition for the PV task, but a significant decrease in activity when DD was anticipated for the remember/ignore task (t(26)=2.85, p=0.008) compared to the ND condition (condition*task interaction: F(1,26)=14.28, p<0.001). Similarly, during the delay period there was again a significant interaction between condition and task (F(1,26)=4.65, p=0.040), which was this time driven by a significant increase between ND and DD conditions for the PV task (t(26)=3.76, p<0.001) but no significant difference between ND and DD conditions for the remember/ignore task (t(26)=0.19, p=0.853).

For RPPA (Figure S7a), the interaction between condition and task failed to reach statistical significance for both the encoding period and the delay period (encoding period: F(1,26)=3.43, p=0.075, delay period: F(1,26)=1.27, p=0.270), and there was no significant main effect of task (encoding period: F(1,26)=0.22, p=0.642, delay period: F(1,26)=0.02, p=0.879), but there was significantly greater activity for the DD condition compared to the ND condition for the delay period (F(1,26)=12.30, p=0.002), but not the encoding period (F(1,26)=2.12, p=0.158). However, the pattern was the same as for LPPA, with a significant increase in activity between ND and DD conditions for the PV task for the encoding period (t(26)=-2.15, p=0.041), and the delay period (t(26)=-3.07, p=0.005), but no significant difference between ND and DD conditions for the remember/ignore task (encoding period: t(26)=0.57, p=0.572, delay period: t(26)=-1.61, p=0.119).

For RFFA (Figure S8a) and LFFA (Figure S8b) there was no significant interaction between condition and task for either time period (encoding period: RFFA: F(1,26)=0.09, p=0.765, LFFA: F(1,26)=0.19, p=0.667, delay period: RFFA: F(1,26)=0.12, p=0.728, LFFA: F(1,26)=0.06, p=0.802). For the encoding period there was no significant difference between ND and DD conditions (RFFA: F(1,26)=0.03, p=0.868, LFFA: F(1,26)=0.33, p=0.569), but for the delay period there was greater activity for the DD condition than the ND condition (RFFA: F(1,26)=20.71, p<0.001), LFFA: F(1,26)=11.82, p=0.002) for both the PV (RFFA: t(26)=2.93, p=0.007, LFFA: t(26)=2.75, p=0.011) and remember/ignore tasks (RFFA: t(26)=3.75, p<0.001, LFFA: t(26)=2.37, p=0.026).

For RFFA and LFFA, during the encoding period there was greater activity for the ignore task compared to the PV task, which reached statistical significance for LFFA (F(1,26)=25.41, p<0.001, ND condition: t(26)=-3.09, p=0.005, DD condition: t(26)=-3.22, p=0.003) but not RFFA (F(1,26)=4.01, p=0.056). There was no significant difference between tasks for the delay period (RFFA: F(1,26)=0.81, p=0.376, LFFA: F(1,26)=0.07, p=0.789).

Direct comparison between ED and DD

Finally, we directly compared task-irrelevant stimulus activity for ND and the two distraction conditions (ED and DD) at the point at which distractors were displayed (the encoding period for ED and the delay period for DD). There was no significant interaction between ROI, time period, task and whether or not distraction was present (F(2.23,57.94)=0.82, p=0.487; Greenhouse-Geisser was used as W(5)=0.49, p=0.004).

As reported above, for both PPA ROIs and both types of distraction there was significantly greater activity for the distraction condition compared to the ND condition for the PV task but this was reduced or absent for the remember/ignore task, at the point at which the distractors were displayed, and this did not significantly differ by distraction type (there was no significant interaction between time period, condition (with or without distraction) and task, RPPA: F(1,26)=0.44, p=0.511, Figure S7a, LPPA: F(1,26)=0.71, p=0.406, Figure S7b).

As mentioned above, for RFFA (Figure S8a) and LFFA (Figure S8b), and for the encoding period, there was significantly greater activity for the distraction condition compared to the ND condition for the PV task but not the remember/ignore task. This had also been observed for both PPA ROIs, but for FFA, unlike PPA, this effect was specific to the encoding period (ED) and not seen for the delay period (DD). Instead, for the delay period, there was an equivalent increase in FFA activity for the DD compared to the ND condition for both PV and remember/ignore tasks, as reported above. Our direct comparison between ED and DD identified that for RFFA, but not LFFA, the two time periods significantly differed (there was a significant interaction between time period, condition and task, for RFFA: F(1,26)=6.34, p=0.018, but not LFFA: F(1,26)=3.80, p=0.062), revealing a significant difference between ED and DD for RFFA.

Discussion

We observed significantly greater encoding period activity for remember compared to ignore conditions for all ROIs except LFFA, exactly corresponding to previous work [1]. For the delay period, results were inconsistent between ROIs, with either equivalent or significantly *lower* activity for the remember compared to the ignore task.

By comparing the WM task to stimulus-matched PV baseline conditions, we probed the extent to which this top-down modulation resulted from enhancement of task-relevant or suppression of task-irrelevant activity. For both types of distraction, we observed significant enhancement in PPA, but not FFA, which was confined to the encoding period. Faces are known to capture attention more rapidly and reliably than other types of stimuli [2,3], which may explain the absence of enhancement when remembering face stimuli compared to PV.

For the delay period, activity associated with the remember task was either equivalent to or *lower* than activity associated with PV (ie. for RPPA and both ED and DD), giving no evidence of enhancement during the delay period. For both PPA ROIs we observed equivalent enhancement (compared to PV) for all three distraction conditions (ND, ED and DD), which was confined to the encoding period. In the delay period, activity in target-relevant regions was not affected by the presence or nature (ED or DD) of distractors and was not elevated compared to stimulus-matched PV conditions, and this was the case for all ROIs.

For all ROIs, instead of observing reduced activity for ignore compared to stimulus-matched PV conditions (suppression), we observed only equivalent activity between these two conditions or *greater* activity for the ignore task compared to PV, although there was a trend for suppression in RFFA (p=0.104).

For ED, regardless of hemisphere, we observed an expected significant increase in PPA activity when scenes were added to the display during the PV task, and a significant increase in FFA activity when faces were added to the display during PV task. However, there was no significant increase in this activity (or, in the case of RPPA this increase was reduced) when the same stimuli were added during the WM task, and should be ignored. This interaction between condition and task was significant for all ROIs except RPPA. This same effect was observed for DD, during the delay period, but only for PPA. For FFA there was an equivalent and significant increase when face distractors were added during the delay period for both PV and WM tasks. When we directly compared the effects of adding ED during the encoding period to the effects of adding DD during the delay period, we observed that ED and DD did not significantly differ for PPA regions, but for FFA they did, supporting our conclusion that participants were not closing their eyes to avoid seeing the DD.

Increases in activity in PPA/FFA when scenes/faces are respectively displayed are robust (Kanwisher et al., 1997, Puce et al., 1995, Epstein & Kanwisher, 1998), and seen in our data for the PV task. We propose that the significant attenuation of that increase under WM task conditions, when those scenes/faces were task-irrelevant, represents a potential mechanism of distractor resistance. For FFA and face distractors shown during the delay period, this mechanism of distractor-resistance appeared to break-down, and FFA activity increased compared to ND for both PV and WM tasks, which may again be associated with more rapid and reliable attentional capture associated with face stimuli [2, 3]. Furthermore, our performance data showed that face DD significantly impaired WM performance whereas face ED and scene distractors did not. Unexpectedly, performance was higher with face ED compared to the ND condition, perhaps indicating an over-compensation for increased task difficulty associated with face ED, perhaps involving the distractor-resistance mechanism we observed.

For LPPA (both time periods) there was a significant increase in task-irrelevant stimulus activity when the task-relevant stimuli should be remembered compared to PV, despite the absence of any task-irrelevant stimuli within the display (the ND condition). This also reached statistical significance for LFFA (only the encoding period), and highlights that, in the absence of overt distraction, top-down modulation is not exclusive to regions associated with task-relevant stimuli (PPA when remembering scenes and FFA when remembering faces), but indicates a more wide-spread boost in activity when stimuli should be remembered. Importantly this was not seen when ED or DD were present, for any ROI. As expected, activity in both PPA ROIs was higher when scenes were added to the screen during PV, but there was no further boost to this activity when the face stimuli should be remembered compared to PV. Similarly, activity in both FFA ROIs was higher when faces were added to the screen during PV, but there was no further boost to this activity when the scene stimuli should be remembered compared to PV. Therefore, this cross-over effect (enhancement of PPA activity when remembering faces and enhancement of FFA activity when remembering scenes) was seen for both LPPA and LFFA, but was absent when distractors were included, and may contribute to mechanisms supporting distractor resistance.

In summary, in line with previous findings [1], results were generally consistent across PPA ROIs. For both PPA ROIs, significant enhancement was seen during the encoding period, which did not differ by condition. Importantly across both PPA ROIs, and for both ED and DD, there was no evidence of reduced activity associated with ignoring scenes compared to PV (ie. suppression). When stimuli were added to the display during PV conditions, their associated activity increased, and this was consistent across all ROIS. However, in the context of a WM task, when those additional stimuli were ED to be ignored, there was no increase seen for LPPA, and both FFA ROIs. There was no significant task * condition * ROI, indicating that this effect did not significantly differ between ROIs (although for RPPA there was an increase when faces should be remembered and scenes should be ignored, which just reached significance). For DD, and scene distractors, this pattern was observed across both PPA ROIs, but for face distractors, FFA activity significantly increased when faces were displayed both for PV and the task in which faces should be ignored, perhaps suggesting a break down in the effectiveness of this mechanism for distractor resistance. Therefore, we generally saw consistency between PPA ROIs, but for FFA, there was an absence of enhancement when faces should be remembered compared to PV, and an increase in activity when faces were presented during the delay period, irrespective of whether they should be ignored, perhaps due to a tendency for face stimuli to capture attention regardless of task demands.

1, Gazzaley, A., Cooney, J.W., McEvoy, K., Knight, R.T., D’Esposito, M. (2005) Top-down enhancement and suppression of the magnitude and speed of neural activity. *J Cog Neuro*, 17, 507-517.

2, Morrisey, M.N., Hofrichter, R., Rutherford, M.D. (2019) Human faces capture attention and attract first saccades without longer fixation. Visual Cognition, 27(3), 1-13.

3, Hershler, O., Hochstein, S. (2005) At first sight: a high-level pop out effect for faces. Vision Research, 45(13), 1707-24.
